# Genomic Epidemiology of Vancomycin-Resistant *Enterococcus faecium* (VR*Efm*) in Latin America: Revisiting The Global VRE Population Structure

**DOI:** 10.1038/s41598-020-62371-7

**Published:** 2020-03-27

**Authors:** Rafael Rios, Jinnethe Reyes, Lina P. Carvajal, Sandra Rincon, Diana Panesso, Aura M. Echeverri, An Dinh, Sergios-Orestis Kolokotronis, Apurva Narechania, Truc T. Tran, Jose M. Munita, Barbara E. Murray, Paul J. Planet, Cesar A. Arias, Lorena Diaz

**Affiliations:** 1 Molecular Genetics and Antimicrobial Resistance Unit, International Center for Microbial Genomics, Universidad El Bosque, Bogotá, Colombia; 2 Center for Antimicrobial Resistance and Microbial Genomics, McGovern Medical School, University of Texas Health Science Center, Houston, TX USA; 3 Division of Infectious Diseases, Department of Internal Medicine, McGovern Medical School, University of Texas Health Science Center, Houston, Texas USA; 4 Institute for Comparative Genomics, American Museum of Natural History, New York, NY USA; 5 Department of Epidemiology and Biostatistics, School of Public Health, SUNY Downstate Health Sciences University, Brooklyn, NY USA; 6 Millennium Initiative for Collaborative Research On Bacterial Resistance (MICROB-R), Santiago, Chile; 7 Genomics and Resistant Microbes Group, Facultad de Medicina Clinica Alemana, Universidad del Desarrollo, Santiago, Chile; 8 Department of Pediatrics, Perelman School of Medicine, University of Pennsylvania & Children’s Hospital of Philadelphia, Philadelphia, PA USA; 9 Department of Microbiology and Molecular Genetics, McGovern Medical School, University of Texas Health Science Center, Houston, Texas USA

**Keywords:** Phylogenetics, Bacterial evolution, Infectious-disease epidemiology, Clinical microbiology

## Abstract

Little is known about the population structure of vancomycin-resistant *Enterococcus faecium* (VR*Efm*) in Latin America (LATAM). Here, we provide a complete genomic characterization of 55 representative Latin American VR*Efm* recovered from 1998–2015 in 5 countries. The LATAM VR*Efm* population is structured into two main clinical clades without geographical clustering. Using the LATAM genomes, we reconstructed the global population of VR*Efm* by including 285 genomes from 36 countries spanning from 1946 to 2017. In contrast to previous studies, our results show an early branching of animal related isolates and a further split of clinical isolates into two sub-clades within clade A. The overall phylogenomic structure of clade A was highly dependent on recombination (54% of the genome) and the split between clades A and B was estimated to have occurred more than 2,765 years ago. Furthermore, our molecular clock calculations suggest the branching of animal isolates and clinical clades occurred ~502 years ago whereas the split within the clinical clade occurred ~302 years ago (previous studies showed a more recent split between clinical an animal branches around ~74 years ago). By including isolates from Latin America, we present novel insights into the population structure of VR*Efm* and revisit the evolution of these pathogens.

## Introduction

Enterococci are predominantly non-pathogenic gastrointestinal commensal bacteria that occasionally cause human infections. Among them, *Enterococcus faecalis* and *Enterococcus faecium* represent the species that account for most clinically relevant infections. In particular, *E. faecium* has been able to adapt to the hospital environment, emerging during the last few decades as a leading cause of health-care infections worldwide and becoming the most challenging enterococcal species to treat^1,2^.

Genome plasticity, the presence of multiple antibiotic resistance determinants and the lack of therapeutic options have contributed to the adaptation of *E. faecium* to hospital environments^3,4^. Moreover, high recombination rates and the acquisition of mobile elements in the genome of *E. faecium* also have driven this evolutionary process^5^. In addition, the enrichment of virulence determinants, such as surface proteins and phosphotransferase systems (particularly PTS^clin^, a putative factor found to contribute to the intestinal colonization in a murine model) seems to provide an advantage to the hospital adaptive process^3,6^. Furthermore, functional gene groups, such as those involved in galactosamine metabolism, bile hydrolysis and phosphorus utilization, are also abundant in *E. faecium* clinical strains compared to non-clinical isolates, suggesting that specific metabolic factors have also facilitated adaptation^7^.

In terms of antibiotic resistance, one of the most relevant antibiotic resistance traits acquired by enterococci is resistance to vancomycin due to acquisition of the *van* gene clusters^8^. Furthermore, vancomycin-resistant *E. faecium* (VR*Efm*) frequently exhibits resistance to ampicillin and high-level resistance to aminoglycosides^9,10^. Indeed, the World Health Organization (WHO) has categorized VR*Efm* as a priority agent for which the finding of new and effective therapeutic strategies is imperative^11^. VR*Efm* is widely distributed in hospitals around the world, with the prevalence varying according to geographical location. In US hospitals, VR*Efm* is an important clinical pathogen, particularly in immunosuppressed and critically-ill patients^1,12^. According to the National Health-Care Safety Network, 82% of *E. faecium* recovered from bloodstream infections in the US were vancomycin-resistant, whereas only 9.8% of *E. faecalis* were resistant to vancomycin^12^. In Europe, prevalence rates of VR*Efm* vary widely by country, but according to the European Centre for Disease and Control (ECDC) 2018 report, overall prevalence (population weighted) has been increasing across European countries, from 10% in 2015 up to 17.3% in 2018^13^. Although data regarding VR*Efm* in Latin America are scarce, a few studies have shed light on the current situation. A prospective multicentre study focusing on 4 countries in northern South America (i.e. Colombia, Ecuador, Peru and Venezuela) found an overall prevalence of VR*Efm* in clinical enterococcal isolates of 31%^14^. More recently, another study performed in Brazil reported an increase in the frequency of isolation of VR*Efm* (over 60%) among infections caused by vancomycin-resistant enterococci between 2007–2015^15^.

Tracking the population structure of *E. faecium* using conventional bacterial typing techniques has been challenging^16^. Although wide genetic variability has been observed among *E. faecium* strains causing clinical infections, a previously described lineage (designated clonal complex CC17 by multi-locus sequence typing [MLST]), was initially recognized as globally distributed^17^. However, the classification of this lineage by MLST has some important drawbacks when analysing the population structure of *E. faecium*. Indeed, the high rates of recombination in MLST loci^18^, the lack of *pstS*^19^ in some strains, and the identification of major discrepancies between MLST compared to whole-genome sequencing (WGS) have limited the accuracy of MLST for typing purposes^20^.

Whole-genome-based comparative phylogenomic analyses using *E. faecium* recovered from different geographical regions have identified two clades, designated A and B. Clade A mostly contains isolates recovered in clinical settings (including those from CC17)^21^, while clade B encompasses organisms isolated in community settings, usually from healthy individuals^3,20,22–24^. A further subdivision has been described within clade A, which groups isolates from animal origin in a subclade (designated as A2), separating them from those recovered from human infections or colonization (subclade A1)^3^.

However, these analyses have been performed using mostly US and European isolates, lacking geographical diversity, particularly in areas such as Latin America. Indeed, studies on the molecular epidemiology of VR*Efm* isolates from Latin America are sparse, with one study suggesting that the CC17 lineage predominates^14^. Furthermore, studies analysing the population structure of VR*Efm* in the region using high-resolution, WGS-based phylogenomic comparative methods are limited. Here, we sought to characterize the population structure of VR*Efm* lineages in a collection of isolates recovered between 1998–2015 in prospective multicentre studies performed in selected Latin-American hospitals^14,25,26^. Using the Latin American genomes, we revisit the global population structure and evolutionary history of VR*Efm*.

## Results

### Genomic characterization of Latin American VR*Efm* clinical isolates

**Figure 1:**
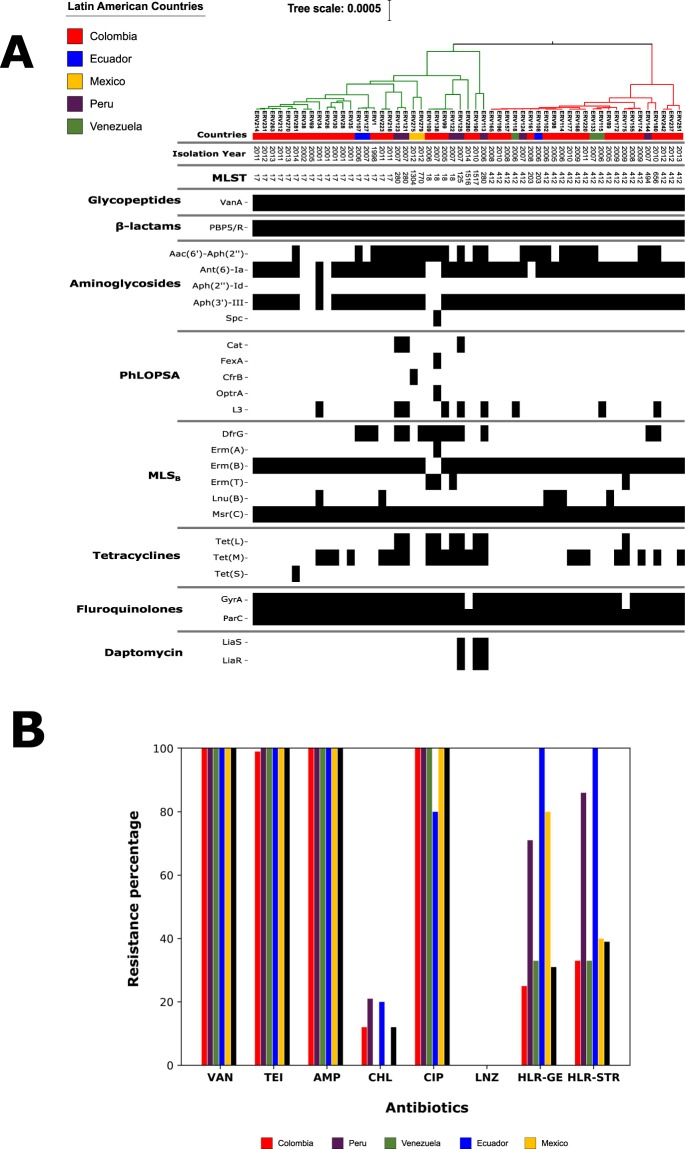
(**A**) Bayesian phylogenomic tree from the core genome and genomic characterization of resistance elements of 55 representative Latin American VR*Efm* strains. The presence of a genetic element is marked by a black box in the corresponding column of the isolate. (**B**) Phenotypic resistance profile of 207 clinical isolates of VR*Efm* from our Latin American collection for vancomycin (VAN), teicoplanin (TEI), ampicillin (AMP), chloramphenicol (CHL), ciprofloxacin (CIP), linezolid (LNZ), high-level resistance to gentamicin (HLR-GE) and high-level resistance to streptomycin (HLR-STR).

From a collection of 207 VR*Efm* clinical isolates obtained between 1998 and 2015 in five Latin American countries (Colombia, Ecuador, Venezuela, Peru and Mexico), we selected 55 representative isolates for WGS. We included the first VR*Efm* (ERV1) reported in Colombia as the representative of 23 isolates with identical PFGE banding pattern, recovered from an outbreak in 1998–1999 and affecting 23 patients in a single teaching hospital^25^. Five isolates (out of 7 available) were selected from a national surveillance in Colombia during 2001–2002, which included 15 tertiary hospitals among 5 cities^26^ and 16 (out of 35 available) were chosen from a subsequent surveillance study (2006–2008) performed in Colombia, Ecuador, Venezuela and Peru and the selected isolates were chosen based on their different banding patterns^14^. The remaining 33 isolates were obtained from sporadic isolates and outbreaks that occurred in Colombia and Mexico (2002–2014). In order to characterize the VR*Efm* lineages circulating in Latin America, we reconstructed their phylogenetic history based on 1,674 genes (groups of orthologous sequences; hereafter referred to as orthogroups) present in more than 90% of the genome sequences (core genome) from a total of 6735 orthogroups (pan-genome) using a Bayesian approach (Fig. 1A). We observed a split into two main clades (Clade I and Clade II, marked in red and green, respectively). Clade I included all the ST412 isolates, while Clade II had all the ST17 isolates from our sample. We observed that the emergence of VR*Efm* in Colombia was associated with Clade II, including the first VR*Efm* (described in 1998) and representatives from the first national surveillance (2001 to 2002). Additionally, ST412 was reported in 2005, and, since then, ST17 and ST412 seem to be the most prevalent STs in the country. In particular, the representative VR*Efm* isolates of the circulating lineages in Peru collected in a two-year period (2006–2007)^14^ exhibited a clear genomic variability (Fig. 1A,B), which correlates to the previously reported diversity based on PFGE and MLST^14^.

### The resistome and virulome of Latin American VR*Efm*

In order to characterize antibiotic resistance determinants, we built resistome profiles by detecting acquired resistance genes and mutations known to confer resistance to linezolid, ciprofloxacin and daptomycin. All the VR*Efm* isolates from our collection were resistant to vancomycin (MIC_90_ > 256 µg/ml) and teicoplanin (MIC_90_ 64 µg/ml) (Fig. 1B). The presence of *vanA* and *vanB* was investigated in all isolates by PCR assays. Consistently, we confirmed the presence of the entire *vanA* cluster in 54 out of the 55 sequenced genomes. Of note, the genome of ERV69 lacked the two-component regulatory system *vanSR*, although it still exhibited MICs of >256 µg/ml and 64 µg/ml for vancomycin and teicoplanin, respectively. Deletion of the genes encoding the two-component regulatory system VanRS has been previously reported^27^.

High-level resistance to ampicillin (MIC > 32 µg/mL) was consistently found in all 55 *E. faecium* isolates, a phenotype that was corroborated using comparisons of the PBP5 protein sequence using a machine-learning prediction model. This approach was based on the amino acid changes present in the PBP5 protein across susceptible and resistant isolates (see details in Methods).

High-level resistance to gentamicin (MIC > 500 µg/mL) was identified in 31% of the isolates of our collection and, within the sequenced representatives, the presence of *aac(6´)-aph(2”)* was detected in 49% of the genome sequences. High-level resistance to streptomycin (MIC > 2000 µg/mL) was identified in 39% of the Latin American VRE*fm* isolates with a high prevalence of the *ant(6)-Ia* gene (89%; n = 49) in the sequenced genomes.

Fluoroquinolone resistance is very common in *E. faecium*. Indeed, all isolates in our collection were fluoroquinolone-resistant and we were able to predict the presence of amino acid substitutions in GyrA and ParC associated with this phenotype. The most common substitution in GyrA was Ser84Arg (67%; n = 37). All isolates exhibited Ser82Arg (53%; n = 29) or Ser82Ile (47%; n = 26) substitutions in ParC.

The *cat* gene, conferring resistance to chloramphenicol, was present only in three Peruvian genomes. All the isolates from our collection were susceptible to linezolid. However, the *optrA* gene was detected in one genome from a Colombian, linezolid-susceptible, isolate (ERV138). We also identified *cfrB*, a recently reported variant of *cfr*^28^, in a Mexican isolate (ERV275). We predicted tetracycline resistance due to *tetM* (43.6%; n = 24), *tetL* (16.3%; n = 9) and *tetS* (1.8%; n = 1) in the sequenced genomes, but resistance to this group of antibiotics was not tested phenotypically. Substitutions in LiaS (Thr120Ala) and LiaR (Trp73Cys), which have been strongly associated with daptomycin resistance and tolerance^29,30^, were present in three VR*Efm* isolates, recovered before daptomycin was available in the region. Of note, the three isolates exhibited MICs between 2–4 µg/ml, considered now as “daptomycin-susceptible dose-dependent”, by the Clinical & Laboratory Standards Institute (CLSI)^31^.

**Figure 2:**
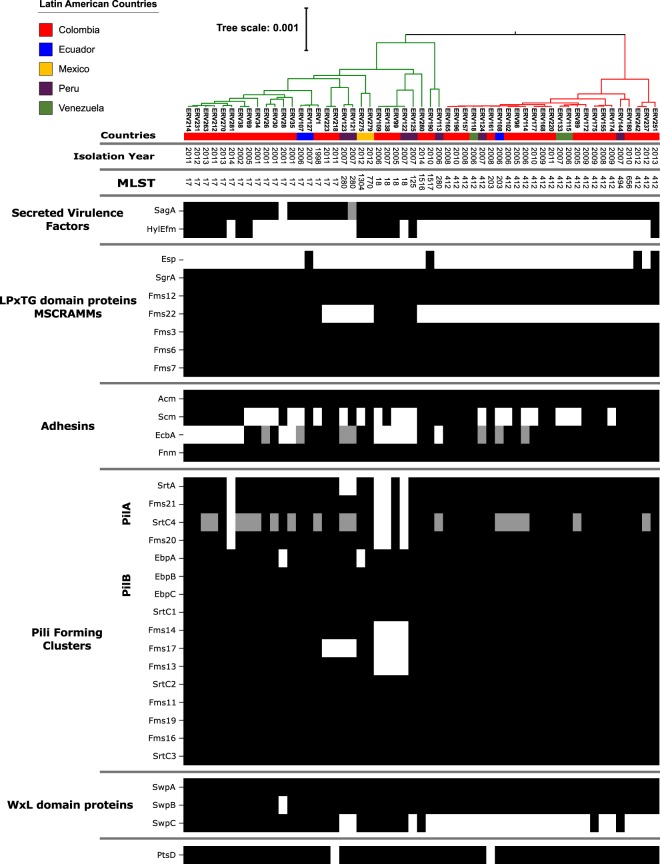
Bayesian phylogenomic tree from the core genome and genomic characterization of virulence factors of 55 representative Latin American VR*Efm* strains, the presence of a genetic element is marked as a black box in the corresponding column, grey boxes show the presence of the genetic element but its sequence has an insertion/deletion compared to the reference sequence.

Latin American VRE isolates also harbored a high proportion of putative virulence determinants (Fig. 2). The vast majority had gene clusters related to pilus formation, adhesins and microbial surface components recognizing adhesive matrix molecules (MSCRAMMS). Interestingly, the notable exception was the Clade I isolates, which often lacked *fms22*, *swpC* and *hyl*_*Efm*_. These results suggest that the “virulome” of our of Latin-American VRE isolates is similar to those from other regions in the world^32^.

### Global phylogenetic reconstructions of latin american VRE

**Figure 3:**
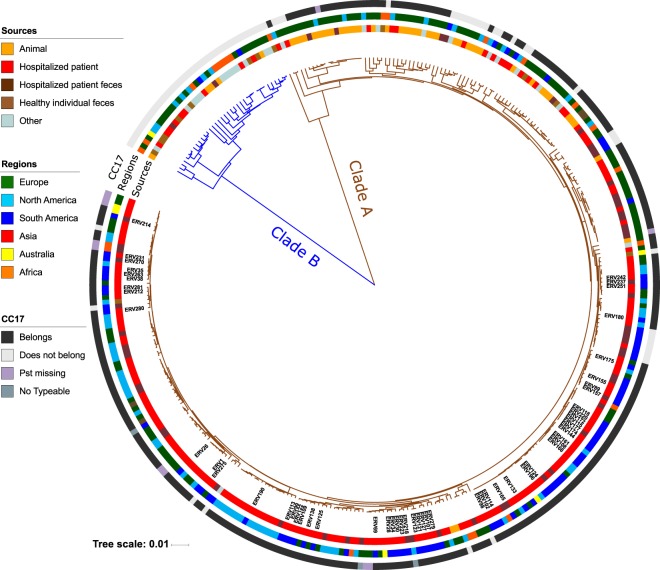
Bayesian phylogenomic tree using the core genome of 340 genomes sampled from 36 countries between 1946 and 2017 and from different sources. Blue branches showed the genomes grouped within clade B, while brown branches show isolates from clade A. The outer coloured rings (from inner to outer) indicate the source of each isolate, the region from which it was sampled and if it was related to Clonal Complex 17. Labels show the isolates originating from our Latin American collection.

To place the genetic lineages of VR*Efm* isolates circulating in Latin America into a global context, we performed a WGS-based phylogenomic analysis. We included 285 *E. faecium* genomes (VRE and non-VRE) from the publicly available NCBI collection aiming to incorporate a diverse set of sequences for comparisons. The included isolates were from colonizing, commensal, animal and clinical sources and were collected between 1946–2017 from Europe, North America, Asia, Africa and Australia (Supplementary Table 1). We constructed a pangenome (29,503 orthogroups) and core genome (978 orthogroups). Using the core genome, we built a phylogenomic tree of the species to show the evolutionary relationships among isolates based on the variation of their genomic sequences. Figure 3 shows that, as previously reported, we found a clear split into two main clades corresponding to the previously designated clades A and B^3,22,24^. All Latin American isolates from our clinical collection were in clade A. We compared the genomic characteristics among the two main clades and found similar findings to a previous publication (Supplementary Table 2 and Supplementary Fig. 1)^3^.

Considering the relevance of *E. faecium* as a cause of hospital-associated infections and that all Latin American isolates were grouped within clade A, we sought to dissect the population structure of this clade. Our first approach was based on a core genome (>90% reconstruction), which contained 1,226 orthogroups and the isolate Com15, from clade B, as the outgroup to root the tree. We observed two major clades. The first clade was composed of 52 genomes, most of which were from animal sources (57%, n = 30), and corresponds to the previously described subclade A2^3^. The second lineage harboured 273 genomes, with 91% (n = 228) corresponding to isolates obtained from clinical sources (Supplementary Fig. 2A), and related to the previously described subclade A1^3^.

Previous studies have shown contradictory distributions of the subclades A1 and A2 within clade A^20^; suggesting that clade A2 is not, in fact, a clade, but rather the paraphyletic early branching lineages of clade A. To further clarify the issue, we performed a Bayesian phylogenomic analysis accounting for recombination events within clade A. We used the variants found from paired alignments of each genome against the chromosome of reference Aus0085 and built a whole-genome multiple sequence alignment (WGMSA) of all genomes in the clade. We used this alignment to create a maximum likelihood (ML) tree, which is required for determining recombinant regions using ClonalFrameML^33^. The average amount of recombination found in the 303 genomes belonging to clade A was 19,539 bp (Supplementary Fig. 2C). The total recombinant regions found across clinical isolates encompassed 1.6 Mb (54% of the length of WGMSA). The exclusion of recombinant regions considerably altered the structure of the tree, and showed 7 early-branching subclades that included 73 genomes (mostly from animal sources) rather than a split into clades A1 and A2. Following these animal-related early branches, we observed a split into two main subclades (Supplementary Fig. 2B). Overall, these subclades were related to clinical sources, exhibiting high similarity in terms of prevalence of antibiotic resistance and virulence determinants (Supplementary Table 3). We refer to them as clinically-related subclades I and II (CRS-I and CRS-II), containing 101 and 124 genomes respectively. Besides the results from the Bayesian analysis, we attempted to obtain support for the topology of the best ML tree used as a guide. We performed 1000 bootstrap resamplings with the non-recombinant matrix. The ML tree showed relatively strong support for the paraphyly of animal-related genomes, and poor support of subclades within the A1 clinical associated clade (Supplementary Fig. 3). Bayesian analysis posterior probability supports were high throughout the tree and supported the monophyly of CRS-I and CRS-II.

**Figure 4:**
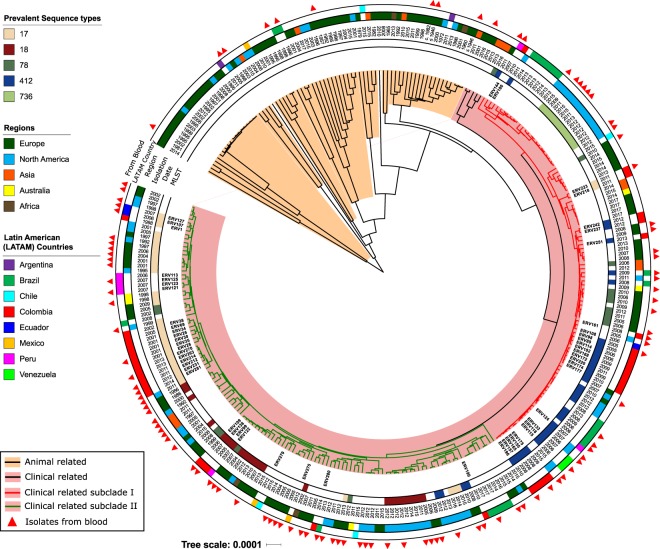
Bayesian phylogenomic tree from non-recombinant regions of 303 Clade A genomes. Branches highlighted in orange represent genomes from animal early branches. Branches highlighted in pink show genomes from clinical related isolates. Red and green branches show the genomes from clinically related subclades (CRS) I and II, respectively. Annotation rings (from inner to outer) show the sequence type (ST) of the isolate (only the five most prevalent STs in the sample are shown), the isolation year, the region from which the isolate was sampled and the exact country from where it was recovered if the source region was Latin America. The last ring shows isolates recovered from blood.

Latin American genomes from our collection were split between these two CRS, showing that Clade I and Clade II (derived from the analysis of Latin American VR*Efm*, see above) belonged to CRS-I and CRS-II, respectively. Of note, the genomes from our collection were distributed almost equally between CRS-I (49%) and CRS-II (51%). Furthermore, despite the inclusion of a few outbreak isolates and that VR*Efm* from Latin America originated in different periods, cities and countries, our phylogenetic reconstruction showed 11 conserved clusters with four or more isolates from the same country (Fig. 4). In particular, three clusters had only Colombian genomes with the number of SNP differences ranging between 36 and 160 within the non-recombinant regions. We also found clusters among isolates from Brazil (n = 3), USA (n = 3), Denmark (n = 1) and Sweden (n = 1). The Danish cluster is situated in the animal-associated branches, and these genomes were closely related (with an average difference of 43 SNPs among them). Of note, two of the USA clusters were related to each other and to 5 other isolates, four of them from the UK and one from Colombia in our collection (172 SNPs average difference).

In CRS-I, there were 23 different STs, with ST412 and ST78 being the most frequent STs (34% and 11%, respectively) (Fig. 4). Importantly, we did not observe a good correlation between MLST and the phylogenomic analysis, as some isolates belonging to the same ST were not all clustered in the same clades, and were distributed in different groups in the phylogeny. In particular, 56% (n = 9) of genomes from ST78 were in CRS-I, while 37% (n = 6) were in CRS-II. To further dissect this discrepancy, we performed a phylogenetic reconstruction using only the sequences of the 7 MLST loci and compared it against the phylogeny of Clade A. Our results showed that many isolates from ST17, ST18, ST78, ST203, ST412 clustered separately from other isolates with the same STs and even formed subclades in the reconstruction that excluded recombinant regions (Supplementary Fig. 4).

In relation to antibiotic resistance determinants, we compared the presence/absence of genomic elements associated with antibiotic resistance between the CRSs and the animal branches (using X^2 ^test on proportions). The animal-associated branches exhibited a lower frequency of elements associated with glycopeptide (34.2%), aminoglycoside (21.9%), ampicillin (9.5%) and fluoroquinolone resistance (2.7%) compared to the CRS isolates, which harboured these determinants in 78%, 85%, 95% and 99% of isolates, respectively. In contrast, similar frequencies of determinants coding for resistance to macrolides (>98%), tetracyclines (between 50–63%) and oxazolidinones (between 2–12%) were found between animal and clinical clades (Supplementary Table 3). Within the subclades of clade A, only 9% of isolates within the animal-associated branches exhibited predicted resistance to ampicillin (7 out of 71 complete PBP5 sequences), while 99% of the clinically related subclades (100% in CRS-I and 98% in CRS-II) were predicted to be resistant^34,35^. Mutations associated with fluoroquinolone resistance were also much more highly prevalent in clinical clades (>98% for CRSs) vs animal branches (2.7%; p < 0.001).

Genes encoding putative surface adhesin proteins (e.g., *acm, scm, esp, sgrA, fms6* and *fms22*) and two of the pilus-forming clusters were significantly more common in the CRSs, (p-values < 0.001 in all cases) compared to animal isolates (Supplementary Table 3). We also compared the presence/absence of putative mobile elements between animal branches vs. CRSs. On average, the number of families of insertion sequences in the former were 5.7, whereas the clinical subclades had 6.9 (6.76 CRS-I and 7.06 for CRS-II). Of note, *rep17* was strongly overrepresented in the CRSs (Supplementary Table 3), located in the plasmid pRUM, which is a representative member of rep17 family and has been associated with the toxin/antitoxin system Txe/Axe^36^.

### Rates of evolution across the whole population of *E. faecium*

Using the sampling date of isolates within clade A, we performed molecular clock analyses on the entire clade A and its subgroups (animal branches, CRS-I and CRS-II). We found that the most recent common ancestor in clade A likely occurred ~2,765 years ago (y.a.) (95% High Posterior Density Interval [HPDI]: [2211, 3372]). The separation of the clinical subclades from the animal branches is predicted to have occurred ~502 y.a. (95% HPDI: [400, 614]) (Supplementary Fig. 5). The most recent split between CRS-I and CRS-II was dated ~302 y. a. (95% HPDI: [227, 348]) (Supplementary Fig. 5). The substitution rate across the clade A genomes was 1.218E-6 (95% HPDI: [1.11E-6, 1.32E-6]) substitutions per site per year, which translates to 3.41 SNPs per genome per year. The substitution rates within each subgroup of genomes were 2.85E-7 (95% HPDI: [2,78E-7, 2.92E-7]) substitutions per site per year for animal associated branches, 4.67E-7 (95% HPDI: [4,1E-7, 4,98E-7]) for CRS-I, and 6.43E-7 (95% HPDI: [5.05E-7, 7.83E-7]) for CRS-II. These rates are equivalent to 0.79, 1.3 and 1.8 SNPs per genome per year, respectively. Our results suggest that clinically related clades seem to evolve faster than those of the animal branches.

## Discussion

Using a multinational collection of strains from Latin America, we provide new insights into the global population structure of VR*Efm*. Unlike previous studies, we found two distinct populations of clinically-related isolates of VR*Efm* when analysing both the Latin American and global isolates. The causes for the splitting of the population structure of VRE (CRS-I and CRS-II) are not clear, but the findings were consistent when analysing the population structure in the presence or absence of recombinant regions. Such a separation suggests that these lineages have been expanding through Latin American countries and highlights the importance of establishing genomic surveillance studies for these multidrug-resistant organisms. Furthermore, the distribution of the Latin American isolates across the tree does not suggest a particular dominance of a specific lineage circulating in the region or country, suggesting that the presence of VR*Efm* in Latin America is likely associated with multiple introductions of VR*Efm* lineages that are circulating globally. Interestingly, some South American countries such as Brazil (no isolates available for this study) have reported VR*Efm* since 1997^37^, and their prevalence appears to be increasing, exhibiting a shift from *E. faecalis* to VR*Efm* since 2007^15^. Of interest, ST412 isolates reported in some regions of Brazil^38,39^ have also been detected in Caribbean countries^40^ and this sequence type was also identified in our collection in Colombia, Peru and Venezuela since 2005^14^, suggesting wide dissemination of this genetic lineage in the region.

Our VR*Efm* phylogenomic analysis, which includes a highly diverse sample collection and excludes recombinant regions from the genome, questions the presence of a single animal clade. If our rooting strategy is correct, our results suggest that the animal isolates represent multiple lineages that diverged prior to the emergence of the clinical subclades in the clade A^3^. Importantly, animal-associated branches have significantly lower ampicillin resistance, mutations associated with fluoroquinolone resistance, virulence determinants, and average number of insertion sequences, similar to what has previously reported^41^. Furthermore, the amount of recombination that we found in clade A genomes was greater than previous results. Importantly, this difference (54% vs 44% found in previous studies^18,42^) could be due to the fact that previous analyses were based on the alignment of SNPs from a core genome and did not include non-coding regions or invariant sites to identify recombinant DNA. Over the recombinant regions, we found partial sequences in 5 out of the 7 loci used by MLST (*ddl, gyd, purK, gdh* and *adk*), corroborating the notion that the current *E. faecium* MLST scheme has major limitations for describing the population structure of VR*Efm*. The exclusion of recombinant regions considerably altered the structure of the tree, dissolving the animal-related clade into a paraphyletic group and reducing the length of the branches across the tree (Supplementary Fig. 1). The discrepancy between MLST and the phylogenomic reconstruction is likely explained by the presence of recombinant regions in the MLST genes and low variation in some of the loci^19,20,43^.

Previous studies estimated that the separation between clades A and B occurred 2776 ± 818 y.a.^3^, a time frame that is similar to our results as the most recent common ancestor (MRCA) of clade A is in line with this date. Because our tree splits clade A2 into a paraphyletic group we expected that the divergence between the last branching animal associated clade and the clinical clade A1 might be even more recent than previously estimated 74 ± 30 y. a^3^. In contrast, we estimated a more ancient split of 502 y.a. (460–546), and, at least, a tenfold lower mutation rate than the estimates in Lebreton *et al*.^3,18^. This finding could be due to the larger genomic region used in our analysis or the additional diversity of the sampled genomes. Our attempts to reproduce the Bayesian analysis divergence times in Lebreton *et al*.^3^, using our dataset but limited to the 50 taxa of clade A in that study, showed even older dates than those presented here (Supplementary Fig. 6). This discrepancy will require additional studies and detailed analysis of sequence change in these lineages that will need to include more systematic sampling over shorter amounts of time. Estimates of rates over the whole species, or in close genus relatives^44^ may also give more context.

The discrepancy between the ML and Bayesian topologies and low bootstrap support within the clinical-related A1 clade, suggests that the split into CRS-I and CRS-II may not be completely accurate, and more data would be required for a precise description of the primary divisions in this clade.

One limitation of our study is the small sample size of genomes from Latin America. We attempted to include representative and diverse strains from our collection based on phenotypic characteristics and PFGE typing of the strains, but further sequencing and sampling may be necessary. Also, we included all publicly available genomes from the region, provided that the associated demographic information was complete (source, year of sampling and geographical location), which emphasizes the low number of previously sequenced genomes of *E. faecium* in Latin America at the moment of sample selection.

## Conclusions

We provide comprehensive insights into the genomic epidemiology of VR*Efm* using available isolates from Latin America where previous studies are lacking. Our results suggest that the population structure of VR*Efm* in the region is diverse and may be grouped into two main lineages (Clades I and II) that belong to the previously reported clade A. Overall, we have presented here a new global reconstruction of *E. faecium*, that uses a wide and diverse sample of isolates from 36 countries. This dataset represents clinical, animal, environmental and commensal samples, and corroborates previous reports that recombination plays a major role in the evolution of this species. Our analyses also indicate, contrary to previous results, that animal-associated genomes are not monophyletic, and are instead a diverse collection of early-branching clades that diverged prior to the emergence of the human clinical clade, at a time that appears to be considerably older than previous estimates. The complex evolutionary dynamics of VR*Efm* highlight the importance of employing phylogenomic approaches when studying the population structure of this highly evolved hospital-associated pathogen.

## Methods

### *Enterococcus faecium* isolates

A total of 207 vancomycin-resistant *E. faecium* clinical isolates from Latin American hospitals recovered between 1998 and 2014 were included in the study. The isolates encompass the first outbreak of VRE infections in Colombia and strains collected in two multicentre hospital surveillances in the region^14,25,26^. Isolates were recovered from patients in Colombia (n = 177, 86%) Peru (n = 14, 7%), Venezuela (n = 6, 3%), Ecuador (n = 5, 2%) and Mexico (n = 5, 2%). The most common sources included blood (22%), urine (18%) and stools (10%). For all isolates, species confirmation (*E. faecium*) was performed by PCR^45^. Antimicrobial susceptibility testing was performed using an agar dilution method^31^.

### Whole genome sequencing

We selected 55 representative isolates from our VR*Efm* collection based on PFGE banding patterns. We included the first VRE reported in Colombia as the representative of an outbreak of 23 infections that occurred at a teaching hospital between 1998 and 1999^25^. Five isolates were selected from a national surveillance in Colombia during 2001–2002, which included 15 tertiary hospitals in 5 cities^26^ and 16 were chosen from subsequent surveillance study performed in Colombia, Ecuador, Venezuela and Peru in 2006–2008^14^. The remaining 33 isolates were sent to our lab for confirmation of resistance or for characterization of outbreaks between 2005–2014. All selected isolates were recovered from clinical samples including blood (32%), urine (13%), faeces (13%), surgical wound (10%), pleural liquid (5%), peritoneal liquid (5%) and other sources (22%). The isolates were subjected to whole genome sequencing on the IIlumina platform. Briefly, genomic DNA was extracted from overnight cultures using the kit DNeasy Blood & Tissue Kit (Qiagen) after a lysozyme treatment. DNA libraries were prepared using Nextera XT kit (illumina) and sequenced on a MiSeq instrument using a 300pb paired-end strategy. The obtained paired-end reads were trimmed for quality using Trimmomatic v0.36^46^, the process included clipping NexteraXT illumina adapters (values 2:30:10 for seedMismatches: palindromeClipThreshold: simpleClipThreshold), followed by trimming of the first and last nucleotides with Q score lower than 3, a sliding window quality check with window size of 4 and an average Q score of 15 and cropping the 2 first nucleotides of the read. Reads lower than 30 nucleotides were discarded. The trimmed reads were used for assemblies using SPAdes v3.13^47^.

### Global *E. faecium* genomic characterization

To place the population structure of Latin American VR*Efm* into global context, we included 285 *E. faecium* genomes from the publicly available collection available at NCBI. We aimed to incorporate a diverse set of sequences, including colonizing, commensal, animal and clinical sources recovered between 1946 and 2017 in Europe, North America, Asia, Africa, and Australia (Supplementary Table 1). the *E. faecium* genomes were grouped into different categories based on source, as follows: (***i***) isolates from stools or rectal swabs from hospitalized patients (n = 59), (***ii***) isolates from hospitalized patients (n = 196), recovered from sources other than faeces, including blood (n = 113), urine (n = 18) and other sources (n = 65), (***iii***) stools from healthy individuals not in hospital settings (n = 13), (***iv***) animal isolates (n = 47), obtained from different animals, including pets, wild and farm animals, and (***v***) “others” (n = 25), which included isolates recovered from food products, water, soil, among other non-human and non-animal sources.

All sequences (340 *E. faecium* genomes) were annotated using RAST^48^. The sequence type (ST) was determined by MLST tools (https://github.com/tseemann/mlst) and verified against PubMLST^49^. Genomic characterization was performed to identify genetic elements associated with resistance using BLASTX^50^ searches against the ResFinder database^51^. Additionally, we specifically interrogated the genomes for amino acid changes in GyrAB and ParCE proteins associated to fluoroquinolone resistance, and mutations in genes encoding 23S rRNA and L3 and L4 proteins associated with linezolid resistance. Detection of mobile elements was performed with BLASTN^50^. Search for *rep* families genes^52,53^ and identification of insertion sequences (IS) was carried out with BLASTN searches and compared to the ISFinder database^54^. Identification of virulence elements was performed with BLASTX against a set of potential virulence proteins in enterococci^4,55^. Identification of CRISPR and *cas-*systems was performed using CRISPRfinder^56^ and BLASTX searches using Cas system proteins^57^, as templates. All BLASTX hits were selected if they had an identity percentage higher or equal to 95% and a coverage of at least 80% of the target sequence. For BLASTN searches, hits were selected if they had an identity percentage higher than 90% and a coverage of at least 80% of the target sequence. To identify statistically significant differences across proportions of the evaluated characteristics among pairs of clades found, a non parametric X^2^ -test was performed (α = 0.01) using the prop.test function from the R programming language^58^.

### Ampicillin resistance prediction based on penicillin-binding protein 5 (PBP5) sequences

To detect ampicillin resistance in *E. faecium*, we developed a random forest model built upon a dataset of 250 PBP5 sequences from isolates with known ampicillin MICs (62 from susceptible isolates [MIC ≤ 8 µg/ml] and 188 belonging to resistant ones [MIC ≥ 16 µg/ml] [Supplementary Table 4]). The model was based on a multiple sequence alignment using the sequence of the PBP5 from Com15 (GenBank accession: WP_002314979.1) isolate as reference (based on previous studies of correlation of the amino acid sequence of this protein with the resistant phenotype^34,35^) encompassing 110 positions harbouring amino acid changes (Supplementary Table 4). These positions were used to create a random forest model with 100 decision trees, using a training set of 42 isolates (17 susceptible and 25 resistant with a range of MIC values). Based on this training set, 40 amino acid changes were selected for the classification based on their discriminatory power applying a recursive elimination process of those with lower score. Next, the model was tested on the whole dataset of PBP5 sequences and had a 100% specificity with 96% sensitivity, which resulted in 6 cases of major errors where the isolate was resistant but predicted to be susceptible.

### Phylogenetic analysis

#### VRE from Latin America

We estimated a Maximum Clade Credibility (MCC) tree in BEASTv1.8.4^59^. From the core genome of 55 representative genomes from our collection of VR*Efm* isolates. We included the genome of isolate Com15 to root the tree. To obtain the core genome we used Roary^60^ and each of the orthogroups was aligned with MUSCLE v3.8^61^. We built a Maximum Likelihood (ML) guide tree with RAxML 8.2.11^62^ using a GTR + Γ model and then rooted it based on Com15. The MCC (0.5 posterior clade probability cut-off and mean heights) tree was constructed employing a constant population size, a GTR + Γ + I substitution model, a strict clock, constant population size, default prior probability distributions, and a chain length of 100 million steps with a burn-in of 10 million and a 5000-step thinning obtaining ESS numbers above 1500.

#### *E. faecium* global population structure

To analyse the global population of *E. faecium*, we estimated a MCC tree including 340 genomes from diverse dates, sources and geographical places (described above) and two outgroups (*Enterococcus durans* BDGP3 [GenBank accession: CP022930.1] and *Enterococcus hirae* ATCC 9790 [CP003504.1]). This tree was based on the core genome (genes present in at least 90% of the studied genomes) obtained with Roary, each orthogroup was individually aligned with MUSCLE and then concatenated to obtain a matrix. The alignment matrix was used for Bayesian phylogenetic reconstruction with BEAST. Model parameters were constant population size, a GTR + Γ + I substitution model, a strict clock, default prior probability distributions, chain length of 300 million steps, a burn-in of 150 million steps (obtaining ESS numbers higher than 4000), and a random starting tree. The final tree was rooted accordingly to the outgroups. The MCC was calculated with 0.5 posterior clade probability cut-off and mean heights.

#### Clade A structure

To study the population structure of *E. faecium* Clade A, we first detected and excluded recombinant regions from a SNP based matrix against the Aus0085 (CP006620.1) reference genome. Subsequently, we obtained a MCC tree, followed by a molecular clock analysis.

To detect the recombinant regions, we performed pairwise comparisons of the 303 genomes grouped in clade A and the Com15 genome against Aus0085 using Mummer 3.23^63^. The identified variants and the reference sequence were used to create a multiple whole genome alignment (mixing the sequence of the Aus0085 genome with the variants from each sample) and building a guide tree with RAxML^62^ using a GTR + Γ model. Subsequently, we rooted the tree based on the sequence of Com15. The branch derived from Com15 was removed and the distance of the root was edited. This guide tree was used to obtain the recombinant regions in the alignment with ClonalFrameML^33^ for each isolate. Those regions were further removed from the alignment to obtain a non-recombinant matrix, which was used to produce a MCC (0.5 posterior clade probability cut-off and mean heights) tree with BEAST. Model parameters were constant population size, a GTR + Γ + I substitution model, a strict clock, default prior probability distributions, chain length of 300 million steps, a burn-in of 50 million steps (obtaining ESS numbers higher than 400), and a guiding starting ML tree from the non-recombinant matrix obtained with RAxML. Additional 1000 bootstraps were calculated for the non-recombination matrix using RAxML and used to give support of the ML guide tree for the Bayesian analysis.

#### Molecular clock analyses

For the molecular clock analyses, we dated the tips of the genomes according to the sampling year. The analysis was performed with the SNPs from the non-recombinant regions of the whole genome alignment as matrix and the previous MCC tree as guide. Model parameters were constant population size, a GTR + Γ + I substitution model, a strict clock, chain length of 300 million steps, a burn-in of 30 million steps, a guiding starting ML tree from the non-recombinant SNPs matrix obtained with RAxML, and default prior probability distributions, except for clock rate (mean = 1E-6, [1E-8, 1E-5]), alpha= 3.30039, nucleotide frequencies of: 0.200, 0.298, 0.299 and 0.203, and substitutions rates for AC = 0.953965, AG = 4.135968, AT = 1.185423, CG = 0.173457 and GT = 4.108113. The obtained ESS numbers were all above 145.

To analyse the molecular clock of isolates of clade A from Lebreton, *et al*. 2013. We used the same SNP based approach as described above, limited to 50 genomes from that study. Model parameters were constant population size, a GTR + Γ + I substitution model, a strict clock, chain length of 300 million steps, a burn-in of 30 million steps, a guiding starting ML tree from the non-recombinant SNPs matrix obtained with RAxML, and default prior probability distributions, except for clock rates (mean = 1E-6, [1E-8, 1E-5]) nucleotide frequencies: 0.208, 0.290, 0.297, 0.205, alpha: 14.258389 and substitutions rates AC = 0.975379, AG = 4.809123, AT = 1.076962, CG = 0.164412 and GT = 4.917563.

To estimate the evolution rates across subclades, further subgrouping of the isolates was performed including animal branches, CRS-I and CRS-II subclades. Subsequently, a similar molecular clock analysis was performed for each group without a guide tree using constant population size, a GTR + Γ + I substitution model, a strict clock, default prior probability distributions, 100 million chain length and 10% burn in (obtaining ESS numbers higher than 200). The three MCC trees were computed with a 0.5 posterior clade probability cut-off and mean heights. All BEAST runs were performed on the CIPRES Science gateway servers^64^.

### Ethics declarations

We declare no ethical competing interest. In our study, we did not perform any experiments with animals or higher invertebrates, neither performed experiments on humans and/or the use of human tissue samples. Our data have been originated from bacteria, not linked to clinical information, collected in previous studies and following ethical approvals. Also, additional genomic data that were included for the analysis are available on public repositories (NCBI and published articles).

## Supplementary information

Supplementary material.

## Data Availability

All genomic data are available at GenBank database, accession numbers for the sequenced genomes are listed in Supplementary Table 3. The datasets generated during and/or analysed during the current study are available from the corresponding author on reasonable requests.
