# Supplementary material for: Genomic Epidemiology of Vancomycin-Resistant Enterococcus faecium (VREfm) in Latin America: Revisiting The Global VRE Population Structure
Source: Sci Rep. 2020 Mar 27;10:5636. doi: 10.1038/s41598-020-62371-7 (PMC7101424; doi:10.1038/s41598-020-62371-7)
Supplement: Supplementary file 1 — Supplementary material. [file 41598_2020_62371_MOESM1_ESM.pdf]

Supplementary material: Genomic Epidemiology of Vancomycin-Resistant *Enterococcus faecium* (VR*Efm*) in Latin America: Revisiting The Global VRE Population Structure

Rafael Rios[1], Jinnethe Reyes[1,2], Lina P. Carvajal[1], Sandra Rincon[1], Diana Panesso[1,2,3], Aura M. Echeverri[1], An Dinh[2,3], Sergios-Orestis Kolokotronis[4,5], Apurva Narechania[4], Truc T. Tran[2,3], Jose M. Munita[2,3,6,7], Barbara E. Murray[2,3,9], Paul J. Planet[4,8], Cesar A. Arias[1,2,3,6,9] and Lorena Diaz[1,2,6*]

[*]Correspondence

Lorena Diaz, PhD

Molecular Genetics and Antimicrobial Resistance Unit, International Center for Microbial Genomics, Universidad El Bosque

Av Cra 9 131a-02, Bogotá, Colombia.

Tel: 57 6489000 ext 1131

email: diazsandra@unbosque.edu.co

Supplementary Table 1. List of *E. faecium* genomes included in the analysis

GENOME	SOURCE CATEGORY	SOURCE	COUNTRY	YEAR	ST	SUBCLADE	ACCESSION
**ICIS96**	Animal	*Equus caballus*	Russia	2014	47	Animal branches	NNSQ01
**E1630**	Other	River water	Netherlands	1981	69	Animal branches	AHXK01
**E3346**	Hospitalized patient	Human - Blood	Netherlands	2002	69	Animal branches	AHYA01
**E0679**	Animal	Pig	Belgium	1995	150	Animal branches	AHWM01
**E0680**	Animal	Pig	Germany	1995	151	Animal branches	AHWN01
**9930238_2**	Animal	Pig feces	Denmark	1999	133	Animal branches	AITJ01
**841V03**	Animal	Pig feces	Denmark	2003	147	Animal branches	AISU01
**E0688**	Animal	Pig	Spain	1995	5	Animal branches	AHWO01
**HF50104**	Animal	Pig	USA	2008	5	Animal branches	AITR01
**E1552**	Hospitalized patient feces	Human feces	Netherlands	2002	5	Animal branches	AHWW01
**1_EFCM_CA_NL**	Hospitalized patient feces	Human feces	Netherlands	2012	6	Animal branches	NXOY01
**E8SV3**	Animal	Pig feces	Denmark	1995	6	Animal branches	AITM01
**7430166_3**	Animal	Pig feces	Denmark	2002	6	Animal branches	AIST01
**9730219_1**	Animal	Pig feces	Denmark	1997	6	Animal branches	AISY01

**7330519_3**	Animal	Pig feces	Denmark	2001	6	Animal branches	AISQ01
**9830091_5**	Animal	Pig feces	Denmark	1998	6	Animal branches	AITC01
**HF50106**	Animal	Pig feces	USA	2008	6	Animal branches	AITT01
**7230532_1**	Animal	Pig feces	Denmark	2000	New ST	Animal branches	AISN01
**A17SV1**	Animal	Pig feces	Denmark	1995	6	Animal branches	AITL01
**IQ110**	Other	Cheese	Argentina	2011	SLV-52	Animal branches	LKPG01
**1231501**	Hospitalized patient	Human - Blood	USA	2005	52	Animal branches	ACAY01
**E1258**	Hospitalized patient	Human - Blood	Spain	1998	127	Animal branches	AHWT01
**5209**	Other	Chicken carcass rinse	USA	2003	54	Animal branches	NSDA01
**7C8_DIV0219**	Animal	Marmot feces	Afghanistan	2015	New ST	Animal branches	NGKZ01
**F1213D01**	Animal	*Bos taurus* feces	Canada	2004	SLV-955	Animal branches	MJDZ01
**D**	Other	Cheese	Mexico	2008	1046	Animal branches	MEGX01
**VAN335**	Animal	Chicken feces	Denmark	2010	417	Animal branches	AIUZ01
**E2071**	Animal	Poultry	Denmark	2001	27	Animal branches	AHXT01
**E1574**	Animal	Dog	Belgium	1995	27	Animal branches	AHWY01
**E0120**	Hospitalized patient	Human - Ascites	Netherlands	1995	27	Animal branches	AHWI01

**E1185**	Hospitalized patient	Human - Blood	France	1998	26	Animal branches	AHWS01
**E1679**	Hospitalized patient	Human - Vascular catheter	Brazil	1998	114	Animal branches	ABSC01
**10_96A**	Hospitalized patient	Human - Blood	Brazil	1996	281	Animal branches	AXOL01
**E1904**	Hospitalized patient	Human - Urine	Netherlands	2001	210	Animal branches	AHXQ01
**E2134**	Animal	Chicken	Netherlands	2004	12	Animal branches	AHXU01
**LS170308**	Animal	Forest musk deer (*Moschus berezovskii*)	China	2017	30	Animal branches	chromosome: NZ_CP025077.1/ CP025077.1
**FDAARGOS_374**	Hospitalized patient	Human - Skin swab	USA	2015	New ST	Animal branches	PDFH01
**7527**	Other	Chicken carcass rinse	USA	2003	269	Animal branches	NSDB01
**E0269**	Animal	Turkey feces	Netherlands	1996	9	Animal branches	AHWK01
**E1620**	Hospitalized patient	Human - Blood	Netherlands	1957	67	Animal branches	AHXF01
**E0045**	Animal	Chicken feces	United Kingdom	1992	9	Animal branches	AHWH01
**E1575**	Animal	Chicken	Belgium	1995	158	Animal branches	AHWZ01
**E0164**	Animal	Turkey feces	Netherlands	1996	26	Animal branches	AHWJ01
**F9730129_1**	Animal	Chicken	Denmark	1997	245	Animal branches	AITN01
**E4215**	Animal	Chicken	Sweden	2004	310	Animal branches	AHYE01

**2D4_DIV0473**	Animal	Buzzard	Germany	2015	195	Animal branches	NGLQ01
**615**	Other	Environmental - Playground slide	USA	2001	82	Animal branches	NSDE01
**E1627**	Hospitalized patient feces	Human feces	Netherlands	1979	66	Animal branches	AHXJ01
**97_6_S5**	Hospitalized patient feces	Human feces	Chile	2015	822	Animal branches	MRYG01
**WEFA23**	Healthy Individual Feces	Human - Healthy infants	China	2015	66	Animal branches	chromosome: CP021885.1
**E1634**	Other	River water	Netherlands	1982	66	Animal branches	AHXL01
**VAN345**	Animal	Chicken feces	Denmark	2010	38	Animal branches	AIVB01
**E4452**	Animal	Dog feces	Netherlands	2008	266	Animal branches	AEOU01
**E1626**	Hospitalized patient	Human - Stomach fluid	Netherlands	1965	92	Animal branches	AHXI01
**GL3**	Animal	Dromedary milk	Algeria	2013	22	Animal branches	LIHM01
**64_3**	Healthy Individual Feces	Human feces	Germany	1992	21	Animal branches	chromosome: NZ_CP012522.1/ CP012522.1
**L_3**	Other	Probiotic	Russia	2010	619	Animal branches	JRGX01
**9B11_DIV0348**	Animal	Bird	Germany	2015	22	Animal branches	NGKT01
**70_36_8**	Animal	Pig	Denmark	2011	536	Animal branches	JRUG01
**E1622**	Animal	Mouse	Netherlands	1959	104	Animal branches	AHXG01

**E1050**	Healthy Individual Feces	Non-hospitalized individual Feces	Netherlands	1998	92	Animal branches	AHWQ01
**NCTC13169**	Hospitalized patient	Human - Blood	United Kingdom	c. 1982	71	Animal branches	ERR375583
**NCTC7380**	Hospitalized patient	Human - Blood	United Kingdom	≤ 1946	32	Animal branches	ERR375572
**E1071**	Hospitalized patient feces	Human feces	Netherlands	2000	32	Animal branches	ABQI01
**ATCC8459**	Other	Cheese	USA	1972	860	Animal branches	AJAJ01
**CRL1879**	Other	Cheese	Argentina	2011	New ST	Animal branches	AOUK01
**11B6_DIV0698**	Animal	Mollusc	USA	2013	DLV-304	Animal branches	NGMC01
**E1573**	Animal	Turkey feces	Netherlands	1996	21	Animal branches	AHWX01
**E1576**	Animal	Ostrich caecum	South Africa	2001	159	Animal branches	AHXA01
**E1623**	Hospitalized patient	Human - Pus	Netherlands	1960	22	Animal branches	AHXH01
**NCTC7171**	Hospitalized patient	Human - Blood	United Kingdom	≤ 1946	160	Animal branches	ERR375571
**LCT_EF128**	Hospitalized patient	Human - Bronchoalveolar lavage	China	2011	160	Animal branches	AJUP01
**E1578**	Animal	Pig feces	Germany	2001	160	Animal branches	AHXB01
**471**	Hospitalized patient	Human - Blood	Russia	2016	SLV-168		PDFM01
**993**	Hospitalized patient	Human - Wound	Russia	2015	SLV-168		PDLZ01

**E161**	Hospitalized patient	Human - Urine	China	2013	414		JXZA01
**E31**	Hospitalized patient	Human - Bile	China	2013	572		JYCO01
**SC4**	Other	Fodder	China	2016	78		chromosome:CP025425.1
**BSAC_EC2783**	Hospitalized patient	Human - Blood	United Kingdom	2011	412	Clinical subclade I	ERR374675
**ERV144**	Hospitalized patient	Human - Blood	Peru	2007	494	Clinical subclade I	AMAK01
**ERV180**	Hospitalized patient	Human Pleural liquid	Colombia	2010	656	Clinical subclade I	MJFF00000000
**MRSN3418**	Hospitalized patient	Human - Wound	USA	2011	412	Clinical subclade I	LBIM01
**LIM1759**	Hospitalized patient feces	Human feces	Brazil	2014	SLV-896	Clinical subclade I	MVGJ01
**LIM1590**	Hospitalized patient feces	Human feces	Brazil	2013	963	Clinical subclade I	MVGG01
**LIM1546**	Hospitalized patient feces	Human - Rectal swap	Brazil	2013	896	Clinical subclade I	MVGO01
**LIM1586**	Hospitalized patient feces	Human feces	Brazil	2013	896	Clinical subclade I	MVGF01
**LIM918**	Hospitalized patient feces	Human feces	Brazil	2012	896	Clinical subclade I	MVGE01
**LIM4247**	Hospitalized patient feces	Human feces	Brazil	2014	896	Clinical subclade I	MVGH01
**LIM1530**	Hospitalized patient feces	Human - Rectal swap	Brazil	2013	896	Clinical subclade I	MVGD01
**IHC2**	Hospitalized patient	Human - Blood	USA	2013	664	Clinical subclade I	MSHR01
**FDAARGOS_326**	Hospitalized patient feces	Human - jejunal aspirate	USA	2015	736	Clinical subclade I	POVW01

**E39**	Hospitalized patient	Human - Blood	USA	2009	736	Clinical subclade I	chromosome: NZ_CP011281.1/ CP011281.1
**ISMMS_VRE_10**	Hospitalized patient	Human - Blood	USA	2011	736	Clinical subclade I	chromosome: NZ_CP012471.1/ CP012471.1
**ER04120.3A**	Hospitalized patient	Human - Blood	USA	2015	736	Clinical subclade I	chromosome: CP023784.1
**ER03933.3A**	Hospitalized patient	Human - Blood	USA	2015	736	Clinical subclade I	chromosome: CP023780.1
**ISMMS_VRE_5**	Hospitalized patient	Human - Blood	USA	2011	736	Clinical subclade I	chromosome: NZ_CP012454.1/ CP012454.1
**E240**	Hospitalized patient	Human - Uterus cancer	USA	2013	736	Clinical subclade I	chromosome: NZ_CP017792.1/ CP017792.1
**909_EFCM**	Hospitalized patient	Human - Urine	USA	2012	736	Clinical subclade I	JUOK01
**702_EFCM**	Hospitalized patient	Human - Urine	USA	2012	736	Clinical subclade I	JUWL01
**514_EFCM**	Hospitalized patient	Human - Urine	USA	2012	736	Clinical subclade I	JVEC01
**377_EFCM**	Hospitalized patient	Human - Sputum induced	USA	2012	736	Clinical subclade I	JVJM01
**43_EFCM**	Hospitalized patient	Human - Blood	USA	2012	736	Clinical subclade I	JVHJ01
**41_EFCM**	Hospitalized patient	Human - Blood	USA	2012	SLV-736	Clinical subclade I	JVIG01
**97_7_S6**	Hospitalized patient feces	Human feces	Chile	2015	New ST	Clinical subclade I	MRYH01
**BSAC_EC2288**	Hospitalized patient	Human - Blood	United Kingdom	2010	78	Clinical subclade I	ERR374760

**197806558**	Hospitalized patient feces	Human feces	Sweden	2014	192	Clinical subclade I	LNLA01
**VRE_1502382**	Hospitalized patient feces	Human feces	Sweden	2015	1489	Clinical subclade I	LNOT01
**VRE_1400136**	Hospitalized patient feces	Human feces	Sweden	2014	192	Clinical subclade I	LNLL01
**S_1402282**	Hospitalized patient feces	Human feces	Sweden	2014	192	Clinical subclade I	LNLD01
**E13931**	Hospitalized patient feces	Human feces	Sweden	2013	192	Clinical subclade I	LNLB01
**ERV223**	Hospitalized patient	Human - Blood	Colombia	2011	17	Clinical subclade I	MJFL00000000
**ERV218**	Hospitalized patient feces	Human - Rectal swap	Colombia	2011	17	Clinical subclade I	MJFJ00000000
**2014_VREF_114**	Hospitalized patient feces	Human - Rectal swap	South Korea	2014	1421	Clinical subclade I	chromosome: NZ_CP019970.1/ CP019970.1
**2014_VREF_268**	Hospitalized patient feces	Human - Rectal swap	South Korea	2015	1421	Clinical subclade I	chromosome: NZ_CP019992.1/ CP019992.1
**EF_DMG150050 1**	Hospitalized patient	Human - No Data	Australia	2014	1421	Clinical subclade I	chromosome 1:LT603678.1
**VREF001**	Hospitalized patient	Human - Cholecystitis	United Kingdom	2017	1424	Clinical subclade I	PJZU01
**VREF005**	Hospitalized patient	Human - Colon cancer	United Kingdom	2017	1424	Clinical subclade I	PJZQ01
**VREF004**	Hospitalized patient	Human - Colon cancer	United Kingdom	2017	1424	Clinical subclade I	PJZR01
**VREF002**	Hospitalized patient	Human - Cholecystitis	United Kingdom	2017	1424	Clinical subclade I	PJZT01
**ERV242**	Hospitalized patient	Human - Urine	Colombia	2012	412	Clinical subclade I	MJFO00000000

**ERV237**	Hospitalized patient feces	Human feces	Colombia	2012	412	Clinical subclade I	MJFN00000000
**K59_68**	Hospitalized patient	Human - Blood	Norway	2008	203	Clinical subclade I	NMZH01
**BSAC_EC2041**	Hospitalized patient	Human - Blood	United Kingdom	2009	203	Clinical subclade I	ERR375174
**ERV251**	Hospitalized patient	Human - Osteomyelitis	Colombia	2013	412	Clinical subclade I	MJFP00000000
**U_1313438**	Hospitalized patient feces	Human feces	Sweden	2013	DLV-203	Clinical subclade I	LNLE01
**S_1001508**	Hospitalized patient feces	Human feces	Sweden	2010	SLV-192	Clinical subclade I	LNLC01
**VRE1044**	Hospitalized patient	Human - Blood	Sweden	2007	192	Clinical subclade I	JAAL01
**VRE1261**	Hospitalized patient	Human - Blood	Sweden	2008	SLV-192	Clinical subclade I	JAAM01
**C309**	Hospitalized patient	Human - Peritoneal fluid	China	2006	78	Clinical subclade I	AJTW01
**XH877**	Hospitalized patient feces	Human feces	China	2012	203	Clinical subclade I	LXWZ01
**VRE16**	Hospitalized patient	Human - Endocarditis	Brazil	2009	412	Clinical subclade I	LSYW01
**ISMMS_VRE_2**	Hospitalized patient	Human - Blood	USA	2011	656	Clinical subclade I	chromosome: NZ_CP012436.1/ CP012436.1
**LIM559**	Hospitalized patient feces	Human feces	Brazil	2008	412	Clinical subclade I	MXAS01
**AUS0085**	Hospitalized patient	Human - Blood	Australia	2009	203	Clinical subclade I	chromosome: NC_021994.1/ CP006620.1
**E6012**	Hospitalized patient feces	Human - Rectal swap	Latvia	2010	78	Clinical subclade I	AHYK01

**BSAC_EC1359**	Hospitalized patient	Human - Blood	United Kingdom	2006	78	Clinical subclade I	ERR374663
**E6045**	Hospitalized patient feces	Human - Rectal swap	Portugal	2010	78	Clinical subclade I	AHYL01
**4686**	Hospitalized patient	Human - Blood	Russia	2009	192	Clinical subclade I	MQRF01
**24**	Hospitalized patient feces	Human feces	Russia	2012	78	Clinical subclade I	PDMA01
**24_10**	Hospitalized patient	Human - Wound Infection	Russia	2011	78	Clinical subclade I	MVEB01
**U0317**	Hospitalized patient	Human - Urinary tract infection	Netherlands	2005	78	Clinical subclade I	ABSW01
**E2560**	Hospitalized patient	Human - Blood	Netherlands	2006	78	Clinical subclade I	AHYI01
**ERV161**	Hospitalized patient	Human - Blood	Colombia	2008	203	Clinical subclade I	AMAW01
**1231502**	Hospitalized patient	Human - Blood	USA	2005	203	Clinical subclade I	ACAX01
**ERV108**	Hospitalized patient	Human - Urine	Ecuador	2006	203	Clinical subclade I	AMAZ01
**ERV98**	Hospitalized patient	Human - Blood	Colombia	2005	412	Clinical subclade I	MJFY00000000
**ERV89**	Hospitalized patient	Human - Bronchial secretion	Colombia	2005	412	Clinical subclade I	MTRT00000000
**ERV114**	Hospitalized patient	Human - Peritoneal liquid	Colombia	2006	412	Clinical subclade I	AMBB01
**ERV102**	Hospitalized patient	Human - Sputum	Colombia	2006	412	Clinical subclade I	AMAX01
**ERV168**	Hospitalized patient	Human - Surgical wound secretion	Colombia	2009	412	Clinical subclade I	AMAU01

**ERV172**	Hospitalized patient	Human - No data	Colombia	2009	412	Clinical subclade I	MJFB00000000
**ERV220**	Hospitalized patient	Human - No data	Colombia	2011	412	Clinical subclade I	MJFK00000000
**ERV174**	Hospitalized patient	Human - Peritoneal liquid	Colombia	2009	412	Clinical subclade I	MJFC00000000
**ERV177**	Hospitalized patient feces	Human - Rectal swap	Colombia	2010	412	Clinical subclade I	MJFE00000000
**805447_07**	Hospitalized patient	Human - Pleura effusion	Austria	2007	412	Clinical subclade I	LQRS01
**IHC11**	Hospitalized patient	Human - Blood	USA	2007	584	Clinical subclade I	MSIB01
**10_131**	Hospitalized patient	Human - Catheter	France	2010	412	Clinical subclade I	PHKY01
**967_EFCM**	Hospitalized patient	Human - Wound	USA	2012	412	Clinical subclade I	JULR01
**964_EFCM**	Hospitalized patient	Human - Fluid	USA	2012	412	Clinical subclade I	JULU01
**990_EFCM**	Hospitalized patient	Human - Fluid	USA	2012	412	Clinical subclade I	JUKT01
**LIM716**	Hospitalized patient feces	Human feces	Brazil	2008	DLV-478	Clinical subclade I	MVGL01
**LIM1547**	Hospitalized patient feces	Human - Rectal swap	Brazil	2013	478	Clinical subclade I	MVGP01
**LIM554**	Hospitalized patient	Human - Blood	Brazil	2008	412	Clinical subclade I	MVGM01
**LIM563**	Hospitalized patient feces	Human feces	Brazil	2009	412	Clinical subclade I	MVGK01
**LIM699**	Hospitalized patient	Human - Blood	Brazil	2006	412	Clinical subclade I	MXAU01
**LIM695**	Hospitalized patient	Human - Blood	Brazil	2006	478	Clinical subclade I	MXAT01

**ERV124**	Hospitalized patient	Human - Surgical wound secretion	Peru	2007	412	Clinical subclade I	AMAM01
**IHC8**	Hospitalized patient	Human - Blood	USA	2007	412	Clinical subclade I	MSHY01
**IHC13**	Hospitalized patient	Human - Blood	USA	2006	412	Clinical subclade I	MSID01
**ERV133**	Hospitalized patient	Human - Urine	Venezuela	2007	412	Clinical subclade I	MJEY00000000
**ERV118**	Hospitalized patient	Human - Blood	Venezuela	2006	412	Clinical subclade I	MTRU00000000
**ERV115**	Hospitalized patient	Human - Surgical wound secretion	Venezuela	2006	412	Clinical subclade I	MTRV00000000
**463_ANA**	Hospitalized patient	Human - Blood	USA	2015	SLV-750	Clinical subclade I	NETE01
**463_AER**	Hospitalized patient	Human - Blood	USA	2015	750	Clinical subclade I	NETD01
**ERV175**	Hospitalized patient	Human - Urine	Colombia	2009	412	Clinical subclade I	MJFD00000000
**ERV155**	Hospitalized patient	Human - Blood	Colombia	2008	412	Clinical subclade I	MJEZ00000000
**ERV165**	Hospitalized patient feces	Human feces	Colombia	2008	412	Clinical subclade I	AMAV01
**ERV157**	Hospitalized patient feces	Human feces	Colombia	2008	412	Clinical subclade I	MJFA00000000
**ERV196**	Hospitalized patient	Human - Blood	Colombia	2010	412	Clinical subclade I	MJFH00000000
**VRE_1502939**	Hospitalized patient feces	Human feces	Sweden	2015	New ST	Clinical subclade II	LNOW01
**VRE2014_7**	Hospitalized patient feces	Human - Rectal swap	USA	2014	New ST	Clinical subclade II	NLCP01
**VREN1625**	Hospitalized patient	Human - Blood	United Kingdom	2015	80	Clinical subclade II	FXHD01

**VREN1371**	Hospitalized patient	Human - Blood	United Kingdom	2015	80	Clinical subclade II	FXHP01
**ICBEF1B**	Other	Seafood	Brazil	2016	1336	Clinical subclade II	NKYP01
**LIM4275**	Hospitalized patient feces	Human feces	Brazil	2014	792	Clinical subclade II	MVGI01
**ET13_17**	Hospitalized patient feces	Human - Rectal swap	Brazil	2017	New ST	Clinical subclade II	PEJX02
**ET12_17**	Hospitalized patient	Human - Blood	Brazil	2017	New ST	Clinical subclade II	PEJV02
**ET14_17**	Hospitalized patient feces	Human - Rectal swap	Brazil	2017	New ST	Clinical subclade II	PEJY02
**ERV190**	Hospitalized patient	Human - No data	Colombia	2010	1517	Clinical subclade II	MJFG00000000
**BSAC_EC1822**	Hospitalized patient	Human - Blood	United Kingdom	2008	412	Clinical subclade II	ERR374884
**BSAC_EC2499**	Hospitalized patient	Human - Blood	United Kingdom	2010	412	Clinical subclade II	ERR374686
**FDAARGOS_332**	Hospitalized patient feces	Human - Stool sample	USA	2015	17	Clinical subclade II	NJFI02
**VRE2014_195**	Hospitalized patient feces	Human - Rectal swap	USA	2014	17	Clinical subclade II	NLCQ01
**VRE2016_194**	Hospitalized patient feces	Human - Rectal swap	USA	2016	17	Clinical subclade II	NLCT01
**VRE001**	Hospitalized patient	Human - Blood	USA	2013	203	Clinical subclade II	chromosome: NZ_CP018071.1/ CP018071.1
**712_EFCM**	Hospitalized patient	Human - Urine	USA	2012	412	Clinical subclade II	JUWB01
**969_EFCM**	Hospitalized patient	Human - Urine	USA	2012	412	Clinical subclade II	JULP01

**BSAC_EC2198**	Hospitalized patient	Human - Blood	United Kingdom	2009	665	Clinical subclade II	ERR374807
**BSAC_EC1484**	Hospitalized patient	Human - Blood	United Kingdom	2006	665	Clinical subclade II	ERR374754
**1230933**	Hospitalized patient	Human - Blood	USA	2005	18	Clinical subclade II	ACAS01
**ISMMS_VRE_11**	Hospitalized patient	Human - Blood	USA	2011	18	Clinical subclade II	chromosome: NZ_CP016163.1 CP016163.1
**VRE2015_149**	Hospitalized patient feces	Human - Rectal swap	USA	2015	18	Clinical subclade II	NLCR01
**FDAARGOS_138**	Hospitalized patient feces	Human - Stool sample	USA	2014	18	Clinical subclade II	LORO02
**MRSN11639**	Hospitalized patient	Human - Wound	USA	2012	18	Clinical subclade II	LBIN01
**199_EFCM**	Hospitalized patient	Human - Blood	USA	2012	18	Clinical subclade II	JVQH01
**999_EFCM**	Hospitalized patient	Human - Abdominal Fluid	USA	2012	18	Clinical subclade II	JUKJ01
**715_EFCM**	Hospitalized patient	Human - Urine	USA	2012	18	Clinical subclade II	JUVY01
**704_EFCM**	Hospitalized patient	Human - Blood	USA	2012	18	Clinical subclade II	JUWJ01
**VRE_1502913**	Hospitalized patient feces	Human feces	Sweden	2015	117	Clinical subclade II	LNOV01
**E2**	Hospitalized patient	Human - Urine	Spain	2011	117	Clinical subclade II	MPZY01
**1250_EFCM**	Hospitalized patient	Human - Blood	USA	2012	117	Clinical subclade II	JVWZ01
**1232_EFCM**	Hospitalized patient	Human - Blood	USA	2012	117	Clinical subclade II	JVXT01

**1238_EFCM**	Hospitalized patient	Human - Blood	USA	2012	117	Clinical subclade II	JVXN01
**97_3_S3**	Hospitalized patient feces	Human feces	Chile	2015	New ST	Clinical subclade II	MRYF01
**EF_AUS00233**	Healthy Individual Feces	Human feces	Australia	2011	796	Clinical subclade II	chromosome 1: NZ_LT598663.1/ LT598663.1
**18434**	Hospitalized patient	Human - Blood	Russia	2012	78	Clinical subclade II	PDFL01
**ERS1122499**	Hospitalized patient	Human - Blood	Germany	2013	17	Clinical subclade II	FKLC01
**9_F_6**	Hospitalized patient	Human - Blood	Norway	2012	872	Clinical subclade II	NMZI01
**MRSN4777**	Hospitalized patient	Human - Wound	USA	2011	No typea ble	Clinical subclade II	LBIL01
**560_EFCM**	Hospitalized patient	Human - Blood	USA	2012	SLV-923	Clinical subclade II	JVCC01
**VRE1558**	Hospitalized patient	Human - Blood	Brazil	2013	No typea ble	Clinical subclade II	LBCV01
**ERV280**	Hospitalized patient	Human - Urine	Colombia	2014	1516	Clinical subclade II	MJFU00000000
**BSAC_EC1116**	Hospitalized patient	Human - Blood	United Kingdom	2005	202	Clinical subclade II	ERR374680
**BSAC_EC2579**	Hospitalized patient	Human - Blood	United Kingdom	2011	117	Clinical subclade II	ERR374756
**HPH2**	Hospitalized patient	Human - Urinary tract infection	Portugal	2007	125	Clinical subclade II	MBRH01
**E1731**	Hospitalized patient	Human - Blood	Tanzania	2002	18	Clinical subclade II	AHXO01

**ERV275**	Hospitalized patient	Human - No data	Mexico	2012	1304	Clinical subclade II	MJFS00000000
**BSAC_EC1179**	Hospitalized patient	Human - Blood	United Kingdom	2005	1042	Clinical subclade II	ERR374732
**BSAC_EC1142**	Hospitalized patient	Human - Blood	United Kingdom	2005	64	Clinical subclade II	ERR374989
**BSAC_EC1201**	Hospitalized patient	Human - Blood	United Kingdom	2005	1477	Clinical subclade II	ERR374823
**BSAC_EC956**	Hospitalized patient	Human - Blood	United Kingdom	2004	1477	Clinical subclade II	ERR375172
**76**	Hospitalized patient	Human - Skin	Russia	2014	80	Clinical subclade II	PDNS01
**97_19_S17**	Hospitalized patient feces	Human feces	Chile	2015	262	Clinical subclade II	MRYE01
**ERV279**	Hospitalized patient	Human - No data	Mexico	2012	770	Clinical subclade II	MJFT00000000
**2E6_DIV0576**	Animal	Bird	Germany	2015	80	Clinical subclade II	NGLP01
**93**	Hospitalized patient	Human - Skin	Russia	2013	18	Clinical subclade II	PDFN01
**BSAC_EC1514**	Hospitalized patient	Human - Blood	United Kingdom	2007	18	Clinical subclade II	ERR374938
**32**	Hospitalized patient	Human - Burn wound	Russia	2010	18	Clinical subclade II	PDFP01
**ST_18_K073**	Hospitalized patient	Human - Urine	South Africa	2013	18	Clinical subclade II	NXIX01
**607_EFCM**	Hospitalized patient	Human - Fluid	USA	2012	18	Clinical subclade II	JVAH01
**E2883**	Hospitalized patient	Human - Blood	Netherlands	2002	18	Clinical subclade II	AHXX01
**BSAC_EC824**	Hospitalized patient	Human - Blood	United Kingdom	2004	18	Clinical subclade II	ERR374662

**BSAC_EC1651**	Hospitalized patient	Human - Blood	United Kingdom	2007	78	Clinical subclade II	ERR374849
**ERV122**	Hospitalized patient	Human - Surgical wound secretion	Peru	2007	18	Clinical subclade II	AMAO01
**ERV99**	Hospitalized patient	Human - Peritoneal liquid	Colombia	2005	18	Clinical subclade II	AMAQ01
**ERV138**	Hospitalized patient	Human - Blood	Colombia	2007	18	Clinical subclade II	AMBD01
**ERV109**	Hospitalized patient	Human - Blood	Colombia	2006	18	Clinical subclade II	AMBC01
**E1321**	Hospitalized patient	Human - Catheter	Italy	1999	78	Clinical subclade II	AHYF01
**E4389**	Animal	Dog feces	Denmark	2008	78	Clinical subclade II	AHYJ01
**E4453**	Animal	Dog feces	Netherlands	2008	192	Clinical subclade II	AEDZ01
**FDAARGOS_331**	Hospitalized patient feces	Human - Stool sample	USA	2015	18	Clinical subclade II	POVX01
**BSAC_EC832**	Hospitalized patient	Human - Blood	United Kingdom	2004	186	Clinical subclade II	ERR375187
**E1133**	Hospitalized patient feces	Human feces	USA	2001	117	Clinical subclade II	AHWR01
**1231408**	Hospitalized patient	Human - Blood	USA	2005	582	Clinical subclade II	ACBB01
**E0333**	Hospitalized patient	Human - Blood	Israel	1997	80	Clinical subclade II	AHWL01
**VRE2**	Hospitalized patient	Human - Blood	Malaysia	2011	80	Clinical subclade II	LTAA01
**VRER5**	Hospitalized patient	Human - Endocarditis	Malaysia	2011	80	Clinical subclade II	LTBJ01

**TX16_DO**	Hospitalized patient	Human - Blood	USA	1992	18	Clinical subclade II	chromosome: NC_017960.1/ CP003583.1
**E1392**	Hospitalized patient feces	Human feces	United Kingdom	2000	64	Clinical subclade II	AHWV01
**NCTC12202**	Hospitalized patient	Human - Blood	United Kingdom	1986	18	Clinical subclade II	ERR375579
**NCTC12204**	Hospitalized patient	Human - Blood	United Kingdom	1986	18	Clinical subclade II	ERR375585
**ISMMS_VRE_8**	Hospitalized patient	Human - Blood	USA	2011	17	Clinical subclade II	chromosome: NZ_CP012465.1/ CP012465.1
**ERV281**	Hospitalized patient	Human - Blood	Colombia	2014	17	Clinical subclade II	MJFV00000000
**ERV231**	Hospitalized patient	Human - Blood	Colombia	2012	17	Clinical subclade II	MJFM00000000
**ERV212**	Hospitalized patient	Human - Blood	Colombia	2011	17	Clinical subclade II	MTRX00000000
**ERV214**	Hospitalized patient feces	Human feces	Colombia	2011	17	Clinical subclade II	MJFI00000000
**ERV263**	Hospitalized patient	Human - Blood	Colombia	2013	17	Clinical subclade II	MJFQ00000000
**ERV270**	Hospitalized patient feces	Human feces	Colombia	2013	17	Clinical subclade II	MJFR00000000
**ERV28**	Hospitalized patient	Human - Surgical wound secretion	Colombia	2001	17	Clinical subclade II	MTRY00000000
**ERV35**	Hospitalized patient	Human - Blood	Colombia	2001	17	Clinical subclade II	MJFX00000000
**ERV30**	Hospitalized patient	Human - Surgical wound secretion	Colombia	2001	17	Clinical subclade II	MTRW00000000
**ERV26**	Hospitalized patient	Human - Pleural liquid	Colombia	2001	17	Clinical subclade II	AMAT01

**ERV34**	Hospitalized patient	Human - No data	Colombia	2001	17	Clinical subclade II	MJFW00000000
**ERV69**	Hospitalized patient	Human - Fistula	Colombia	2005	17	Clinical subclade II	AMAR01
**ERV38**	Hospitalized patient	Human - No data	Colombia	2002	17	Clinical subclade II	AMAS01
**TX0082**	Hospitalized patient	Human - Blood	USA	1999	17	Clinical subclade II	AEBU01
**LIM714**	Hospitalized patient feces	Human feces	Brazil	2008	New ST	Clinical subclade II	MVGN01
**E1644**	Hospitalized patient	Human - Catheter-Urine	Germany	2002	78	Clinical subclade II	AHYG01
**E2369**	Hospitalized patient	Human - Wound	Hungary	2005	78	Clinical subclade II	AHYH01
**E6**	Hospitalized patient feces	Human feces	Spain	2009	17	Clinical subclade II	MQAC01
**AUS0004**	Hospitalized patient	Human - Blood	Australia	1998	17	Clinical subclade II	chromosome: NC_017022.1/ CP003351.1
**E525**	Hospitalized patient	Human - Wound	Australia	1998	17	Clinical subclade II	AUWV01
**ERV121**	Hospitalized patient	Human - Blood	Peru	2007	280	Clinical subclade II	AMAP01
**ERV123**	Hospitalized patient	Human - Urine	Peru	2007	280	Clinical subclade II	AMAN01
**ERV125**	Hospitalized patient	Human - Blood	Peru	2007	125	Clinical subclade II	AMAL01
**ERV113**	Hospitalized patient	Human - Urine	Peru	2006	280	Clinical subclade II	MJEW00000000
**E155**	Healthy Individual Feces	Human - Stool sample	USA	1995	17	Clinical subclade II	AUWX01

**BSAC_EC239**	Hospitalized patient	Human - Blood	United Kingdom	2001	17	Clinical subclade II	ERR374681
**BSAC_EC227**	Hospitalized patient	Human - Blood	United Kingdom	2001	17	Clinical subclade II	ERR374688
**BSAC_EC754**	Hospitalized patient	Human - Blood	United Kingdom	2004	17	Clinical subclade II	ERR374714
**VRE0576**	Hospitalized patient	Human - Blood	Sweden	2006	17	Clinical subclade II	JAAK01
**E1162**	Hospitalized patient	Human - Blood	France	1997	17	Clinical subclade II	ABQJ01
**ATCC51559**	Hospitalized patient	Human - Blood	USA	1992	17	Clinical subclade II	JSVT01
**E1165**	Hospitalized patient	Human - Wound	Italy	1997	17	Clinical subclade II	AUWW01
**1231410**	Hospitalized patient	Human - Skin and soft tissue	USA	2005	17	Clinical subclade II	ACBA01
**E2297**	Hospitalized patient	Human - Urine	USA	2001	117	Clinical subclade II	AHXV01
**ERV1**	Hospitalized patient	Human - Pleural liquid	Colombia	1998	17	Clinical subclade II	AMAY01
**ERV107**	Hospitalized patient	Human - Blood	Ecuador	2006	17	Clinical subclade II	AMBA01
**ERV127**	Hospitalized patient	Human - Blood	Ecuador	2007	17	Clinical subclade II	MJEX00000000
**VRE3**	Hospitalized patient	Human - Blood	USA	1998	SLV-16	Clinical subclade II	JSET01
**C68**	Hospitalized patient feces	Human feces	USA	1997	16	Clinical subclade II	LPUE01
**BSAC_EC426**	Hospitalized patient	Human - Blood	United Kingdom	2002	16	Clinical subclade II	ERR374669
**BSAC_EC495**	Hospitalized patient	Human - Blood	United Kingdom	2002	16	Clinical subclade II	ERR374844

**1141733**	Hospitalized patient	Human - Wound	USA	2005	327	Clade B	ACAZ01
**17OM39**	Healthy Individual Feces	Human - Stool sample	India	2015	76	Clade B	LWHF01
**7H8_DIV0219**	Animal	Marmot feces	Afghanistan	2015	New ST	Clade B	NGKW01
**825**	Animal	Dairy cattle feces	USA	2004	SLV-94	Clade B	NSDF01
**BM4107**	Hospitalized patient feces	Human feces	France	1988	172	Clade B	JMII01
**BSAC_EC555**	Hospitalized patient	Human - Blood	United Kingdom	2003	1036	Clade B	ERR374785
**COM12**	Healthy Individual Feces	Non-hospitalized individual Feces	USA	2006	107	Clade B	ACBC01
**COM15**	Healthy Individual Feces	Non-hospitalized individual Feces	USA	2007	583	Clade B	ACBD01
**E1007**	Healthy Individual Feces	Non-hospitalized individual Feces	Netherlands	1998	61	Clade B	AHWP01
**E1590**	Healthy Individual Feces	Non-hospitalized individual Feces	Ireland	2001	163	Clade B	AHXC01
**E1604**	Other	Cheese	Norway	1956	75	Clade B	AHXD01
**E1613**	Other	Fish burger	Norway	1964	77	Clade B	AHXE01
**E1861**	Hospitalized patient feces	Human feces	Spain	2001	289	Clade B	AHXP01
**E1972**	Hospitalized patient	Human - Blood	Germany	2000	94	Clade B	AHXR01

**E2039**	Hospitalized patient	Human - K-spitze	Germany	2000	296	Clade B	AHXS01
**E2620**	Hospitalized patient	Human - Blood	Netherlands	2006	1175	Clade B	AHXW01
**E3083**	Hospitalized patient	Human - Blood	Netherlands	2000	327	Clade B	AHXZ01
**E3548**	Hospitalized patient	Human - Blood	Netherlands	2004	328	Clade B	AHYB01
**E980**	Healthy Individual Feces	Human - Healthy volunteer	Netherlands	1998	94	Clade B	ABQA01
**F1129D110**	Animal	Bos taurus feces	Canada	2004	New ST	Clade B	MJDY01
**GM75**	Other	Cheese	Argentina	2011	94	Clade B	LKPI01
**HP_5_10**	Healthy Individual Feces	Human feces	Russia	2016	New ST	Clade B	PCGB01
**JB00008**	Other	Korean fermented soybean paste (cheonggukjang)	South Korea	2015	94	Clade B	NPOO01
**KACC15689**	Other	Doenjang fermented soybean paste	South Korea	2013	New ST	Clade B	LBIT01
**KACC15700**	Other	Ganjang fermented soy sauce	South Korea	2013	New ST	Clade B	LDNC01
**KACC15711**	Other	Meju fermented soybeans	South Korea	2013	New ST	Clade B	LDND01
**KACC15960**	Other	Doenjang fermented soybean paste	South Korea	2013	New ST	Clade B	LDNE01

**KACC16093**	Other	Meju fermented soybeans	South Korea	2013	New ST	Clade B	LDNH01
**L_X**	Other	Probiotic preparation	Russia	2010	812	Clade B	JRGY01
**M3K31**	Animal	Griffon vulture	Spain	2010	76	Clade B	LAXK01
**NCTC7378**	Hospitalized patient	Human - Blood	United Kingdom	≤ 1946	361	Clade B	ERR375567
**PC4_1**	Healthy Individual Feces	Non-hospitalized individual Feces	Australia	2008	720	Clade B	ADMM01
**TX1330**	Healthy Individual Feces	Non-hospitalized individual Feces	USA	2006	107	Clade B	ACHL01
**11F9MSG5001**	Animal	Chelonia mydas feces	Brazil	0	688	Clade B	NGMK01
**UBA4566**	Other	Wood	USA	2015	946	Clade B	DGKQ01
**UC7256**	Other	Food	Italy	0	74	Clade B	AWWM01
**UC8668**	Other	Food	Italy	0	New ST	Clade B	AWWN01

Supplementary table 2. Genomic characteristics of the clades A and B of *E. faecium* population.

	CLADE A	CLADE B
**Genome size**	2.908 Mb ± 0.19 Mb	2.833 Mb ± 0.16 Mb
**Gene content**	2801 ± 208 genes	2736 ± 156 genes
**Pan genome**	10382 orthogroups	23746 orthogroups
**Core genome**	1182 orthogroups	1466 orthogroups
**Average number of virulence genes**	21.6	13.7
**Average number of *rep* plasmids genes**	3.24	2.16
**Average number of Insertion Sequences (IS) families**	7.4	4.1

Supplementary table 3. Comparison of the prevalences and frequencies of genetic elements related to antibiotic resistance, virulence, mobile elements, plasmid families and CRISPR/CAS-system among the genomes from the subgroups in the clade A. Not applicable (NA) results were obtained when the proportion of the element for both compared groups was zero.

**Category**	**Description**	**Element**	**Animal branches (n=73) % (n)**	**CRS-I (n=101) % (n)**	**CRS-II (n=124) % (n)**	**P value of the difference of proportions (X^2 test) between Animal vs CRS-I**	**Animal vs CRS-II**	**95% Confidence Intervals of the differences of proportions Animal vs CRS-I**	**Animal vs CRS-II**
Resistance	Glycopeptides resistance	VanA	32,8 (24)	70,2 (71)	65,3 (81)	< .001	< .001	[-52.6, -22.3]	[-47.2, -17.7]
		VanB	0 (0)	11,8 (12)	9,6 (12)	.005	.014	[-19.4, -4.4]	[-16, -3.4]
		VanD	1,3 (1)	0 (0)	0 (0)	.87	.788	[-2.5, 5.2]	[-2.4, 5.1]
		Total	34,2 (25)	82,1 (83)	75 (93)	< .001	< .001	[-62.3, -33.6]	[-55.1, -26.4]
	Aminoglycosides resistance	aac(6')-aph(2'')	6,8 (5)	52,4 (53)	47,5 (59)	< .001	< .001	[-58.1, -33.1]	[-52.3, -29.1]
		ant(6)-Ia	16,4 (12)	74,2 (75)	71,7 (89)	< .001	< .001	[-71, -44.6]	[-68, -42.6]
		aph(3')-III	12,3 (9)	82,1 (83)	71,7 (89)	< .001	< .001	[-81.6, -58.1]	[-71.5, -47.4]
		aadD, aph(2'')-Ib, aph(2'')-Id, spc or str	15 (11)	7,9 (8)	4 (5)	.212	.013	[-3.8, 18.1]	[1, 21]
		Total	21,9 (16)	90 (91)	81,4 (101)	< .001	< .001	[-80.5, -55.9]	[-72.3, -46.7]
	Fluroquinolones resistance	GyrA mutations	1,3 (1)	96 (97)	86,2 (107)	< .001	< .001	[-100, -88.8]	[-92.6, -77.2]
		ParC mutations	2,7 (2)	100 (101)	86,2 (107)	< .001	< .001	[-100, -92.3]	[-91.8, -75.3]
		Total	2,7 (2)	100 (101)	98,3 (122)	< .001	< .001	[-100, -92.3]	[-100, -90.2]
	Ampicillin resistance	PBP5-R	9,5 (7)	94 (95)	96,7 (120)	< .001	< .001	[-93.8, -75.1]	[-95.7, -78.7]
	Trimethoprim resistance	dfrG	4,1 (3)	13,8 (14)	53,2 (66)	.06	< .001	[-19.1, -0.4]	[-60.1, -38.1]

	MLSb resistance		erm(A)	5,4 (4)	6,9 (7)	3,2 (4)	.942	.688	[-9.8, 6.9]	[-4.9, 9.4]
			erm(B)	39,7 (29)	85,1 (86)	83 (103)	< .001	< .001	[-59.8, -31]	[-57.4, -29.2]
			erm(G)	0 (0)	0 (0)	0,8 (1)	NA	1	[0, 0]	[-3.2, 1.6]
			erm(T)	1,3 (1)	0,9 (1)	10,4 (13)	1	.034	[-3.3, 4.1]	[-16.2, -2]
			lnu(B)	9,5 (7)	25,7 (26)	4,8 (6)	.012	.317	[-28.2, -4.1]	[-4.1, 13.6]
			msr(C)	95,8 (70)	100 (101)	97,5 (121)	.142	.812	[-9.8, 1.6]	[-8.1, 4.7]
			vat(D)	6,8 (5)	0 (0)	0 (0)	.027	.013	[-0.1, 13.8]	[0, 13.7]
			vat(E)	5,4 (4)	0 (0)	0 (0)	.061	.034	[-0.9, 11.9]	[-0.8, 11.8]
			Total	98,6 (72)	100 (101)	100 (124)	.87	.788	[-5.2, 2.5]	[-5.1, 2.4]
	Tetracyclines resistance		tet(L)	19,1 (14)	34,6 (35)	30,6 (38)	.038	.11	[-29.6, -1.3]	[-24.7, 1.8]
			tet(M)	46,5 (34)	44,5 (45)	60,4 (75)	.912	.08	[-14.2, 18.2]	[-29.3, 1.5]
			tet(S)	1,3 (1)	0,9 (1)	3,2 (4)	1	.74	[-3.3, 4.1]	[-7, 3.3]
			Total	50,6 (37)	57,4 (58)	63,7 (79)	.467	.1	[-22.9, 9.4]	[-28.4, 2.3]
	Phlopsa resistance		cat	9,5 (7)	0 (0)	9,6 (12)	.005	1	[1.7, 17.5]	[-8.7, 8.5]
			fexA	0 (0)	0 (0)	0,8 (1)	NA	1	[0, 0]	[-3.2, 1.6]
			cfrB	0 (0)	0 (0)	0,8 (1)	NA	1	[0, 0]	[-3.2, 1.6]
			optrA	0 (0)	0 (0)	0,8 (1)	NA	1	[0, 0]	[-3.2, 1.6]
			23s mutation	0 (0)	2,9 (3)	2,9 (3)	.37	.304	[-7.5, 1.5]	[-7.4, 1]
			Total	9,5 (7)	2,9 (3)	11,2 (14)	.128	.892	[-2.1, 15.3]	[-11.5, 8.1]
Virulence	Pili forming clusters	PilA	SrtA	79,4 (58)	87,1 (88)	85,4 (106)	.369	.229	[-20.2, 4.8]	[-18.3, 6.2]
			Fms21	78 (57)	87,1 (88)	89,5 (111)	.047	.047	[-21.7, 3.7]	[-23.4, 0.6]
			SrtC4	73,9 (54)	81,1 (82)	70,9 (88)	.772	.359	[-21, 6.6]	[-10.9, 16.9]
			Fms20	75,3 (55)	88,1 (89)	91,12 (113)	.004	.007	[-25.7, 0.1]	[-28, -3.6]
		PilB	EbpA	76,7 (56)	97 (98)	97,58 (121)	< .001	< .001	[-31.7, -8.9]	[-32, -9.7]
			EbpB	73,9 (54)	97 (98)	97,58 (121)	< .001	< .001	[-34.8, -11.3]	[-35.1, -12.1]
			EbpC	75,3 (55)	95 (96)	98,38 (122)	< .001	< .001	[-31.6, -7.8]	[-34.3, -11.8]
			SrtC1	83,5 (61)	97 (98)	99,19 (123)	< .001	< .001	[-23.8, -3.2]	[-25.4, -5.9]

Pili forming clusters	Fms14	68,4 (50)	91 (92)	91,12 (113)	< .001	< .001	[-35.8, -9.4]	[-35.5, -9.8]
	Fms17	69,8 (51)	88,1 (89)	87,09 (108)	.005	.007	[-31.7, -4.8]	[-30.4, -4.1]
	Fms13	91,7 (67)	98 (99)	93,5 (116)	.857	.358	[-14.3, 1.8]	[-10.5, 7]
	SrtC2	100 (73)	100 (101)	100 (124)	NA	1	[0, 0]	[0, 0]
	Fms11	30,1 (22)	81,1 (82)	95,96 (119)	< .001	< .001	[-65.2, -36.9]	[-78, -53.7]
	Fms19	27,3 (20)	86,1 (87)	95,96 (119)	< .001	< .001	[-72.2, -45.3]	[-80.5, -56.7]
	Fms16	30,1 (22)	87,1 (88)	96,77 (120)	< .001	< .001	[-70.6, -43.4]	[-78.7, -54.6]
	SrtC3	30,1 (22)	87,1 (88)	96,77 (120)	< .001	< .001	[-70.6, -43.4]	[-78.7, -54.6]
LPxTG-containing proteins Adhesins	Acm	53,4 (39)	100 (101)	99,1 (123)	< .001	< .001	[-59.2, -34]	[-58.4, -33.1]
	Scm	21,9 (16)	56,4 (57)	48,38 (60)	< .001	< .001	[-49.2, -19.8]	[-40.5, -12.4]
	EcbA	4,1 (3)	59,4 (60)	38,7 (48)	< .001	< .001	[-67.1, -43.5]	[-45.4, -23.8]
	Esp*fm*	0 (0)	28,7 (29)	25 (31)	< .001	< .001	[-38.7, -18.7]	[-33.7, -16.3]
	SgrA	6,8 (5)	80,1 (81)	66,93 (83)	< .001	< .001	[-84.2, -62.5]	[-71.3, -48.9]
	Fms3	100 (73)	100 (101)	98,4 (122)	NA	1	[0, 0]	[-1.6, 3.2]
	Fms6	84,9 (62)	100 (101)	99,2 (123)	< .001	< .001	[-24.5, -5.7]	[-24.4, -5.8]
	Fms7	83,6 (61)	96 (97)	95,2 (118)	.021	.013	[-21.3, -0.9]	[-21, -1]
	Fms12	93,2 (68)	98 (99)	97,6 (121)	.408	.273	[-10.6, 3.6]	[-10.6, 2.9]
	Fms22	24,7 (18)	0,9 (1)	58,1 (72)	< .001	< .001	[13.6, 36.5]	[-47.2, -18.5]
	Fnm	100 (100)	98 (99)	97,5 (121)	.625	.461	[-1.9, 5.9]	[-1.4, 6.2]
Secreted antigen A	SagA	87,6 (64)	98 (99)	94,3 (117)	.014	.164	[-19.5, -1.2]	[-16.3, 3]
Carbohydrate transport and gut colonization	PtsD	5,4 (4)	95 (96)	93,5 (116)	< .001	< .001	[-97.5, -81.7]	[-95.9, -80.2]
Glycosyl hydrolase	*hyl*Efm	0 (0)	19,8 (20)	34,6 (43)	< .001	< .001	[-28.8, -10.9]	[-44.1, -25.2]
WxL-containing proteins	SwpA	100 (100)	100 (101)	100 (124)	NA	NA	[0, 0]	[0, 0]
	SwpB	94,5 (69)	97 (98)	98,3 (122)	.659	.273	[-9.9, 4.9]	[-10.6, 2.9]
	SwpC	84,9 (62)	27,7 (28)	91,1 (113)	< .001	.271	[44, 70.4]	[-16.9, 4.5]

Plasmid families	*pLG1* megaplasmid	rep-Efae287	82,1 (60)	85,1 (86)	84,6 (105)	.752	.979	[-15.3, 9.4]	[-14.4, 9.4]
	pRUM	rep17	10,9 (8)	79,2 (80)	62,09 (77)	< .001	< .001	[-80.1, -56.4]	[-63.4, -38.9]
	Rolling circle replication	rep14	6,8 (5)	18,8 (19)	46,77 (58)	.041	< .001	[-22.7, -1.2]	[-51.5, -28.3]
	Rep_3	rep-pCIZ2	6,8 (5)	20,7 (21)	37,9 (47)	.019	< .001	[-24.9, -3]	[-42.5, -19.6]
		rep11	4,1 (3)	28,7 (29)	20,96 (26)	< .001	.002	[-35.7, -13.5]	[-26.4, -7.3]
		rep18	6,8 (5)	28,7 (29)	28,22 (35)	< .001	< .001	[-33.6, -10.1]	[-32.3, -10.5]
	Inc18 (pRE25)	rep2	26 (19)	40,5 (41)	40,3 (50)	.066	.06	[-29.6, 0.5]	[-28.6, 0.1]
		rep1	34,2 (25)	0,9 (1)	10,48 (13)	< .001	< .001	[21, 45.5]	[10.5, 37]
	Broad range hosts	rep7	1,3 (1)	0,9 (1)	4 (5)	1	.534	[-3.3, 4.1]	[-8.1, 2.8]
	Described in *S. aureus*	rep22	5,4 (4)	15,8 (16)	20,16 (25)	.06	.009	[-20.4, -0.4]	[-24.6, -4.8]
		rep-pUB110	8,2 (6)	15,8 (16)	20,16 (25)	.206	.043	[-18.3, 3.1]	[-22.5, -1.4]
	Rep trans	rep-pHTBeta	1,3 (1)	1,9 (2)	14,51 (18)	1	.005	[-5, 3.8]	[-21, -5.3]
Insertion Sequences	IS16	IS16	9,5 (7)	95 (96)	99,1 (123)	< .001	< .001	[-94.6, -76.3]	[-97.6, -81.6]
	IS3	ISefa10, ISEfa8 and ISenfa3	75,3 (55)	96 (97)	99,19 (123)	< .001	< .001	[-32.5, -8.9]	[-35, -12.8]
	IS1182	ISefa12 and ISEfa7	36,9 (27)	89,1 (90)	91,12 (113)	< .001	< .001	[-65.9, -38.3]	[-67.4, -40.9]
	IS200/IS605	ISEfa4 and ISEnfa200	24,6 (18)	90 (91)	74,19 (92)	< .001	< .001	[-78.1, -52.8]	[-63.2, -35.9]
	IS19/IS982	ISEfm1	91,7 (67)	80,1 (81)	90,3 (112)	.057	.93	[0.4, 22.8]	[-7.8, 10.7]
	ISL3	ISEfa11 and ISEfa5	20,5 (15)	82,1 (83)	82,25 (102)	< .001	< .001	[-74.7, -48.6]	[-74.2, -49.2]
	IS6 *Lactococcus lactis*	ISS1	71,2 (52)	83,1 (84)	53,2 (66)	.09	.019	[-25.8, 1.9]	[3.3, 32.7]
	IS6 *Lactobacillus sake*	ISS1W	78 (57)	29,7 (30)	37,09 (46)	< .001	< .001	[34.2, 62.6]	[27.2, 54.8]
	IS256	ISEfa13 and ISEfm2	76,7 (56)	30,6 (31)	43,5 (54)	< .001	< .001	[31.6, 60.4]	[19, 47.3]

IS256 *Lactococcus garvieae*	ISLgar5	26 (19)	6,9 (7)	44,3 (55)	.001	.015	[6.7, 31.5]	[-32.7, -3.9]
IS5	ISKpn26	0 (0)	0,9 (1)	0 (0)	1	NA	[-3.9, 1.9]	[0, 0]
CRISPR		38,3 (28)	23,7 (24)	30,6 (38)	.048	.056	[-0.5, 29.7]	[-7.2, 22.6]
Cas-system		0 (0)	0 (0)	1,6 (2)	1	NA	[0, 0]	[-4.9, 1.7]

PBP5 amino acid positions

			2	3	4	5	17	20	24	25	27	28	29	32	34	39	60	66	67	68	73	85	94	95	100	111	115	141	144	151	159	172	177	204	216	218	226	235	262	265	271	272	276	280	290	295	301	314	317	318	324	325	326	339	344	346	349	350	354	357	358	386	397	398	401	405	406	407	408	414	426	430	434	439	440	451	460	461	462	466	466	470	476	485	496	497	499	509	510	524	525	546	552	553	558	561	562	573	581	582	586	598	601	606	625	626	629	632	642	644	646	663	667	672
Previously described by Pietta et al. AAC. 2014.									X		X				X			X		X		X			X				X			X	X	X	X																X																													X	X			X	X		X				X										X						X						X	
Used in prediction model									X		X				X	X		X		X		X			X				X			X	X	X				X															X														X									X				X	X	X				X	X	X			X		X				X			X	X	X	X						X	X	X		X		X	
	AMP	Accession																																																																																																																		
Com15	0,25	ACBD01000014.1	K	R	S	D	A	V	V	A	S	G	G	Y	R	T	D	G	M	A	A	E	A	D	E	D	S	N	K	P	E	T	L	D	A	D	Q	P	G	Y	A	A	G	D	P	N	S	T	G	K	T	D	G	N	K	T	A	K	T	D	S	D	E	D	A	N	P	E	Q	F	M	A	D	D	P	W	Y	Q	V	-	S	Q	A	M	N	F	A	D	L	E	E	N	A	M	A	G	T	E	I	G	V	Q	N	S	E	K	E	K	P	N	E	T	P	Y
E0120	0,5	AHWI01000003.1	.	.	.	.	.	.	A	.	G	.	.	.	.	.	.	.	.	.	.	.	.	.	Q	.	.	.	Q	.	.	A	.	.	.	.	.	T	.	.	.	.	.	.	.	.	.	.	.	.	.	.	.	.	.	.	.	.	.	.	.	.	.	.	.	.	.	.	.	.	.	.	.	N	.	.	.	.	.	.	.	.	.	.	K	.	T	.	F	.	D	.	.	.	.	.	.	D	V	S	.	.	.	.	.	.	.	.	.	.	G	.	.	.
EnGen0048	0,5	AHYE01000001.1	.	.	.	.	.	.	A	.	G	.	.	.	Q	.	.	E	.	.	.	.	.	.	Q	.	.	.	Q	.	.	A	I	.	S	.	.	.	.	.	.	.	.	.	.	.	.	.	.	.	A	.	.	.	.	.	.	.	.	.	N	.	.	.	S	.	.	.	.	.	.	.	.	.	.	.	.	.	.	.	.	.	.	.	K	.	I	.	.	.	D	.	.	.	.	.	.	.	.	.	.	.	.	.	.	.	.	.	.	.	G	.	.	.
UC7267	0,5	ASAM01000001.1	.	.	.	.	.	.	A	.	G	.	.	.	Q	.	.	E	.	.	.	.	.	.	Q	.	.	.	Q	.	.	A	I	.	S	.	.	.	.	.	.	.	.	.	.	.	.	.	.	.	A	.	.	.	.	.	.	.	.	.	.	.	.	.	S	.	.	.	.	.	.	.	.	.	.	.	.	.	.	.	.	.	.	.	K	.	I	.	.	.	D	.	.	.	.	.	.	.	.	.	.	.	.	.	.	.	.	.	.	.	G	.	.	.
E980	1	ABQA01000020.1	.	.	.	.	.	.	A	.	G	.	.	.	.	.	.	.	.	.	T	.	.	.	.	.	.	.	Q	.	.	.	.	.	.	.	.	.	.	.	.	.	.	.	.	.	.	.	.	.	A	.	.	.	.	.	.	.	.	.	.	.	.	.	.	.	.	.	.	.	.	.	.	.	.	.	.	.	.	.	.	.	.	.	.	.	.	.	.	.	.	.	.	.	.	.	.	.	.	.	.	.	.	.	.	.	.	.	.	.	G	.	.	.
TX1330	1	ACHL01000102.1, ACHL01000101.1, EEI60277	.	.	.	.	.	.	.	.	.	.	.	.	.	.	.	.	.	.	.	.	.	.	.	.	.	.	.	.	.	.	.	.	.	.	.	.	.	.	.	.	.	.	.	.	.	.	.	.	.	.	.	.	.	.	.	.	.	.	.	.	.	.	.	X	X	.	.	.	.	.	.	.	.	.	.	.	.	.	.	.	.	.	.	.	.	.	.	.	.	.	.	.	.	.	.	.	.	.	.	.	.	.	.	.	.	.	.	.	G	.	A	.
E1185	1	AHWS01000017.1	.	.	.	.	.	.	A	.	G	.	.	.	Q	.	.	E	.	.	.	.	.	.	Q	.	.	.	Q	.	.	A	I	.	S	.	.	.	.	.	.	.	.	.	.	.	.	.	.	.	A	.	.	.	.	.	.	.	.	.	.	.	.	.	S	.	.	.	.	.	.	.	.	.	.	.	.	.	.	.	.	.	.	.	K	.	I	.	.	.	D	.	.	.	.	.	.	.	.	.	.	.	.	.	.	.	.	.	.	.	G	.	.	.
E0164	1	AHWJ01000010.1	.	.	.	.	S	.	A	.	G	.	.	.	Q	N	.	E	.	.	.	.	.	.	Q	.	.	.	Q	.	.	A	I	.	S	.	.	.	.	.	.	.	.	.	.	.	.	.	.	.	A	.	.	.	.	.	.	.	.	.	.	.	.	.	.	.	.	.	.	.	.	.	.	.	.	.	.	.	.	.	.	.	.	.	K	.	T	.	.	.	D	.	.	.	.	.	.	.	.	.	.	.	.	.	.	.	.	.	.	.	G	.	.	.
E1627	1	AHXJ01000003.1	.	.	.	.	.	.	A	.	G	.	.	.	Q	N	.	E	.	.	.	.	.	.	Q	.	.	.	Q	.	.	A	I	.	S	.	.	.	.	.	.	.	.	.	.	.	.	.	.	.	A	.	.	.	.	.	.	.	.	.	.	.	.	.	.	.	.	.	.	.	.	.	.	.	.	.	.	.	.	.	.	.	.	.	K	.	T	.	.	.	D	.	.	.	.	.	.	.	.	.	.	.	.	.	.	.	.	.	.	.	G	.	.	.
UC10237	1	AWWP01000001.1	.	.	.	.	.	.	A	.	G	.	.	.	Q	.	.	E	.	T	.	D	.	.	Q	.	.	.	Q	.	.	A	I	G	S	.	.	.	.	.	.	.	.	.	.	.	.	.	.	.	A	.	.	.	.	.	.	.	.	.	.	.	.	.	.	.	.	.	.	.	.	.	.	.	.	.	.	.	.	.	.	.	.	.	K	.	T	.	.	.	D	.	.	I	.	C	.	.	.	.	.	.	.	.	.	.	.	.	.	.	G	.	S	.
E1573	1	AHWX01000001.1	.	.	.	.	.	.	A	.	G	.	.	.	Q	.	.	E	.	T	.	D	.	.	Q	.	.	.	Q	.	.	A	I	G	S	.	.	.	.	.	.	.	.	.	.	.	.	.	.	.	A	.	.	.	.	.	.	.	.	.	.	.	.	.	.	.	.	.	.	.	.	.	.	.	.	.	.	.	.	.	.	.	.	.	K	.	T	.	.	.	D	.	.	.	.	.	.	.	.	.	.	.	.	.	.	.	.	.	.	.	G	.	S	.
UC7256	2	AWWM01000001.1	.	.	.	.	.	.	A	.	G	.	.	.	.	.	.	.	.	.	T	.	.	.	.	.	.	.	Q	.	.	.	.	.	.	.	.	.	.	.	.	.	.	.	.	.	.	.	.	.	A	.	.	.	.	.	.	.	.	E	.	.	G	.	.	.	.	.	.	.	.	.	.	.	.	.	.	.	.	.	.	.	.	.	.	.	.	.	.	.	.	.	.	.	.	.	.	.	.	.	.	.	.	.	.	.	.	.	.	.	G	.	.	.
1141733	2	ACAZ01000019.1	.	.	.	.	.	.	.	.	.	.	.	.	.	.	.	.	.	.	.	.	.	.	.	.	.	.	.	.	.	.	.	.	.	.	.	.	.	.	.	.	.	.	L	.	.	.	.	.	.	.	.	.	.	.	.	.	.	.	.	.	.	.	.	.	.	.	.	.	.	.	.	.	.	.	.	.	.	.	.	.	.	.	.	.	.	.	.	.	.	.	.	.	.	.	.	.	.	.	.	.	.	.	.	.	.	.	.	.	G	.	L	.
UC8733	2	AWWO01000001.1	.	.	.	.	.	.	A	.	G	.	.	.	Q	N	.	E	.	.	.	.	.	.	Q	.	.	.	Q	.	.	A	I	.	S	.	.	.	.	.	.	.	.	.	.	.	.	I	.	.	A	.	.	.	.	.	.	.	.	.	.	.	.	.	.	.	A	.	.	.	.	.	.	.	.	.	.	.	.	.	.	.	.	.	K	.	T	.	.	.	D	.	.	.	.	.	.	.	.	.	.	.	.	.	.	.	.	.	.	.	G	.	.	.
E0680	4	AHWN01000006.1	.	.	.	Y	.	.	A	.	G	.	.	.	Q	.	.	E	.	.	.	.	.	.	Q	.	.	.	Q	.	.	A	I	.	S	.	.	.	.	.	.	.	.	.	.	.	.	.	.	.	A	.	.	.	.	.	.	.	.	.	.	.	.	.	S	.	.	.	.	.	.	.	.	.	.	.	.	.	.	.	.	.	.	.	K	.	I	.	.	.	D	.	.	.	.	.	.	.	.	.	.	.	.	.	.	.	.	.	.	.	G	.	.	.
E0045	4	AHWH01000023.1	.	.	.	.	.	.	A	.	G	.	.	.	Q	.	.	E	.	.	.	.	.	.	Q	.	.	.	Q	.	.	A	I	.	S	.	.	.	.	.	.	.	.	.	.	.	.	.	.	.	A	.	.	.	.	.	.	.	.	.	.	.	.	.	S	.	.	.	.	.	.	.	.	.	.	.	.	.	.	.	.	.	.	.	K	.	I	.	.	.	D	.	.	.	.	.	.	.	.	.	.	.	.	.	.	.	.	.	.	.	G	.	.	.
E1552	8	AHWW01000007.1	.	.	.	.	.	.	A	.	G	.	.	.	Q	.	.	E	.	.	.	.	.	.	Q	.	.	.	Q	.	.	A	I	.	S	.	.	.	.	.	.	.	.	.	.	.	.	.	.	.	A	.	.	.	.	.	.	.	.	.	.	.	.	.	S	.	.	.	.	.	.	.	.	.	.	.	.	.	.	.	.	.	.	.	K	.	I	.	.	.	D	.	.	.	.	.	.	.	.	.	.	.	.	.	.	.	.	.	.	.	G	.	.	.
E1613	≤8	AHXE01000011.1	.	.	.	.	.	.	.	.	G	.	S	.	.	.	.	.	.	.	.	.	.	N	.	.	.	.	.	.	.	.	.	.	.	G	.	H	.	.	.	.	.	.	.	S	.	.	.	.	A	.	.	.	.	.	.	.	.	.	.	.	.	.	.	.	.	.	.	.	.	.	.	.	.	.	.	.	.	.	.	.	.	.	.	.	.	.	.	.	.	.	.	.	.	.	.	.	.	.	.	.	.	.	.	.	.	.	.	.	G	.	.	.
E1861	≤8	AHXP01000002.1	.	.	.	.	.	.	A	.	G	.	.	.	.	.	.	.	.	.	T	.	.	.	.	.	.	.	Q	.	.	.	.	.	.	.	.	.	.	.	.	.	.	.	.	.	.	.	.	R	A	.	.	.	.	.	.	.	.	.	.	.	.	.	.	.	.	.	.	.	.	.	.	.	.	.	.	.	.	.	.	.	.	.	.	.	.	.	.	.	.	.	.	.	.	.	.	.	.	.	.	.	.	.	.	.	.	.	.	.	G	.	.	.
E2620	≤8	AHXW01000008.1	.	.	.	.	.	.	.	.	G	.	.	.	.	.	.	.	.	.	.	.	.	.	.	.	.	.	.	L	.	.	.	.	V	.	.	.	.	.	.	.	.	.	.	.	.	.	.	.	.	.	.	.	.	.	.	.	.	.	.	.	.	.	.	.	.	.	.	.	.	.	.	.	.	.	.	.	.	.	.	.	.	.	.	.	.	.	.	G	.	.	.	.	.	.	.	.	.	.	.	.	.	.	.	.	.	.	.	.	G	.	.	.
BM4107	≤8	AF364092.1	.	.	.	.	.	.	.	T	.	.	.	.	.	S	.	.	.	.	.	.	.	.	.	.	.	.	.	.	.	.	.	.	.	.	.	.	.	.	.	.	.	.	.	.	.	.	.	.	.	.	.	.	.	.	.	.	.	.	.	.	.	.	.	.	.	.	.	.	.	.	.	.	.	.	.	.	.	.	.	.	.	.	.	.	.	.	.	.	.	.	.	.	.	.	.	.	.	.	.	.	.	.	.	.	.	.	.	D	G	.	.	.
E1604	≤8	AHXD01000013.1	.	.	.	.	.	.	.	.	.	.	.	.	.	.	.	.	.	.	.	.	.	.	.	.	.	.	.	.	.	.	.	.	.	.	.	.	.	.	.	.	.	.	.	.	.	.	.	.	.	.	.	.	.	.	.	.	.	.	.	N	.	.	.	.	.	.	.	.	.	.	.	.	.	-	.	.	.	.	.	.	.	.	.	.	.	.	.	.	.	.	.	.	.	.	.	.	.	.	.	.	.	.	.	.	.	.	.	.	G	.	.	.
E3548	≤8	AHYB01000002.1	.	.	.	.	.	A	.	.	.	.	.	.	.	.	.	.	.	.	.	.	.	.	.	.	.	.	.	.	.	.	.	.	.	.	.	.	.	.	.	.	.	.	.	.	.	.	.	.	.	.	.	.	.	.	.	.	.	.	.	.	.	.	.	.	.	.	.	.	.	.	.	.	.	.	.	.	.	.	.	.	.	.	.	.	.	.	.	.	.	.	.	.	.	.	.	.	.	.	.	.	.	.	.	.	.	.	.	.	G	.	.	.
D63r	≤8	X84860.1	.	.	.	.	.	.	.	.	.	.	.	.	.	.	.	.	.	.	.	.	.	.	.	.	.	.	.	.	.	.	.	.	.	.	.	.	.	.	.	.	.	.	.	.	.	.	.	.	.	.	.	.	.	.	.	.	.	.	.	.	.	.	.	.	.	.	.	.	.	.	.	.	.	.	.	.	.	.	.	H	.	.	.	.	.	.	.	.	.	.	.	.	.	.	.	.	.	.	.	.	.	.	.	.	.	.	.	.	G	.	.	.
1231501	≤8	ACAY01000033.1	.	.	.	.	.	.	.	.	.	.	.	.	.	.	.	.	.	.	.	.	.	.	.	.	.	.	.	.	.	.	.	.	.	.	.	.	.	.	.	.	.	.	.	.	.	.	.	.	.	.	.	.	.	.	.	.	.	.	.	.	.	.	.	.	.	.	.	.	.	.	.	.	.	.	.	.	.	.	.	.	.	.	.	.	.	.	.	.	.	.	.	.	.	.	.	.	.	.	.	.	.	.	.	.	.	.	.	.	G	.	.	.
E1007	≤8	AHWP01000007.1	.	.	.	.	.	.	.	.	.	.	.	.	.	.	.	.	.	.	.	.	.	.	.	.	.	.	.	.	.	.	.	.	.	.	.	.	.	.	.	.	.	.	.	.	.	.	.	.	.	.	.	.	.	.	.	.	.	.	.	.	.	.	.	.	.	.	.	.	.	.	.	.	.	.	.	.	.	.	.	.	.	.	.	.	.	.	.	.	.	.	.	.	.	.	.	.	.	.	.	.	.	.	.	.	.	.	.	.	G	.	.	.
E3083	≤8	AHXZ01000002.1	.	.	.	.	.	.	.	.	.	.	.	.	.	.	.	.	.	.	.	.	.	.	.	.	.	.	.	.	.	.	.	.	.	.	.	.	.	.	.	.	.	.	.	.	.	.	.	.	.	.	.	.	.	.	.	.	.	.	.	.	.	.	.	.	.	.	.	.	.	.	.	.	.	.	.	.	.	.	.	.	.	.	.	.	.	.	.	.	.	.	.	.	.	.	.	.	.	.	.	.	.	.	.	.	.	.	.	.	G	.	.	.
E1590	≤8	AHXC01000004.1	.	.	.	.	.	.	.	.	.	.	.	.	.	.	.	.	.	.	.	.	.	.	.	.	.	.	.	.	.	.	.	.	.	.	.	.	.	.	.	.	.	.	.	.	.	.	.	.	.	.	.	.	.	.	.	.	.	.	.	.	.	.	.	.	.	.	.	.	.	.	.	.	.	.	.	.	.	.	.	.	.	.	.	.	.	.	.	.	.	.	.	.	.	.	.	.	.	.	.	.	.	.	.	.	.	.	.	.	G	.	L	.
Com12	≤8	ACBC01000012.1	.	.	.	.	.	.	.	.	.	.	.	.	.	.	.	.	.	.	.	.	.	.	.	.	.	.	.	.	.	.	.	.	.	.	.	.	.	.	.	.	.	.	.	.	.	.	.	.	.	.	.	.	.	.	.	.	.	.	.	.	.	.	.	.	.	.	.	.	.	.	.	.	.	.	.	.	.	.	.	.	.	.	.	.	.	.	.	.	.	.	.	.	.	.	.	.	.	.	.	.	.	.	.	.	.	.	.	.	G	.	A	.
VAN327	≤8	ASEC01000004.1	.	.	.	.	.	.	A	.	G	.	.	.	.	.	.	.	.	.	.	.	.	.	Q	.	.	.	Q	.	.	A	.	.	.	.	.	T	.	.	.	.	.	.	.	.	.	.	.	.	.	.	S	.	.	.	.	.	.	.	.	.	.	.	.	.	.	.	.	.	.	.	.	N	.	.	.	.	.	.	.	.	.	.	K	.	T	.	F	.	D	.	.	.	.	.	.	D	V	S	.	.	.	.	.	.	.	.	.	.	G	.	.	.
VAN335	≤8	AIUZ01000021.1	.	.	.	.	.	.	A	.	G	.	.	.	.	.	.	.	.	.	.	.	.	.	Q	.	.	.	Q	.	.	A	.	.	.	.	.	T	.	.	.	.	.	.	.	.	.	.	.	.	.	.	S	.	.	.	.	.	.	.	.	.	.	.	.	.	.	.	.	.	.	.	.	N	.	.	.	.	.	.	.	.	.	.	K	.	T	.	F	.	D	.	.	.	.	.	.	D	V	S	.	.	.	.	.	.	.	.	.	.	G	.	.	.
E1574	≤8	AHWY01000003.1	.	.	.	.	.	.	A	.	G	.	.	.	.	.	.	.	.	.	.	.	.	.	Q	.	.	.	Q	.	.	A	.	.	.	.	.	T	.	.	.	.	.	.	.	.	.	.	.	.	.	.	.	.	.	.	.	.	.	.	.	.	.	.	.	.	.	.	.	.	.	.	.	N	.	.	.	.	.	.	.	.	.	.	K	.	T	.	F	.	D	.	.	.	.	.	.	D	V	S	.	.	.	.	.	.	.	.	.	.	G	.	.	.
E2071	≤8	AHXT01000013.1	.	.	.	.	.	.	A	.	G	.	.	.	.	.	.	.	.	.	.	.	.	.	Q	.	.	.	Q	.	.	A	.	.	.	.	.	T	.	.	.	.	.	.	.	.	.	.	.	.	.	.	.	.	.	.	.	.	.	.	.	.	.	.	.	.	.	.	.	.	.	.	.	N	.	.	.	.	.	.	.	.	.	.	K	.	T	.	F	.	D	.	.	.	.	.	.	D	V	S	.	.	.	.	.	.	.	.	.	.	G	.	.	.
D366	≤8	X84859.1	-	-	-	-	.	.	A	.	G	.	.	.	Q	.	.	E	.	.	.	.	.	.	Q	.	.	.	Q	.	.	A	I	G	S	.	.	.	.	.	.	.	.	.	.	.	.	.	.	.	A	.	.	.	.	.	.	.	.	.	.	.	.	.	S	.	.	.	.	.	.	.	.	.	.	.	.	.	.	.	.	.	.	.	K	.	I	.	.	.	D	.	.	.	.	.	.	.	.	.	.	R	.	.	.	.	.	.	.	.	G	.	.	.
E1039	≤8	ACOS01000009.1	.	.	.	.	.	.	A	.	G	.	.	.	Q	.	.	E	.	.	.	.	.	.	Q	.	.	.	Q	.	.	A	I	.	S	.	.	.	.	.	.	.	.	.	.	.	.	.	.	.	A	.	.	.	.	.	.	.	.	.	.	.	.	N	S	.	.	.	.	.	.	.	.	.	.	.	.	.	.	.	.	.	.	.	K	.	I	.	.	.	D	.	.	.	.	.	.	.	.	.	.	.	.	.	.	.	.	.	.	.	G	.	.	.
E1634	≤8	AHXL01000003.1	.	.	.	.	.	.	A	.	G	.	.	.	Q	.	.	E	.	.	.	.	.	.	Q	.	.	.	Q	.	.	A	I	.	S	.	.	.	.	.	.	.	.	.	.	.	.	.	.	.	A	.	.	.	.	.	.	.	.	.	.	.	.	.	S	.	.	K	.	.	.	.	.	.	.	.	.	.	.	.	.	.	.	.	K	.	I	.	.	.	D	.	.	.	.	.	.	.	.	.	.	.	.	.	.	.	.	.	.	.	G	.	.	.
841V03	≤8	AISU01000016.1	.	.	.	.	.	.	A	.	G	.	.	.	Q	.	.	E	.	.	.	.	.	.	Q	.	.	.	Q	.	.	A	I	.	S	.	.	.	.	.	.	.	.	.	.	.	.	.	.	.	A	.	.	.	.	.	.	.	.	.	.	.	.	.	S	.	.	.	.	.	.	.	.	.	.	.	.	.	.	.	.	.	.	.	K	.	I	.	.	.	D	.	.	.	.	.	.	.	.	.	.	.	.	.	.	.	.	.	.	.	G	.	.	.
E0679	≤8	AHWM01000004.1	.	.	.	.	.	.	A	.	G	.	.	.	Q	.	.	E	.	.	.	.	.	.	Q	.	.	.	Q	.	.	A	I	.	S	.	.	.	.	.	.	.	.	.	.	.	.	.	.	.	A	.	.	.	.	.	.	.	.	.	.	.	.	.	S	.	.	.	.	.	.	.	.	.	.	.	.	.	.	.	.	.	.	.	K	.	I	.	.	.	D	.	.	.	.	.	.	.	.	.	.	.	.	.	.	.	.	.	.	.	G	.	.	.
E1575	≤8	AHWZ01000016.1	.	.	.	.	.	.	A	.	G	.	.	.	Q	.	.	E	.	.	.	.	.	.	Q	.	.	.	Q	.	.	A	I	.	S	.	.	.	.	.	.	.	.	.	.	.	.	.	.	.	A	.	.	.	.	.	.	.	.	.	.	.	.	.	S	.	.	.	.	.	.	.	.	.	.	.	.	.	.	.	.	.	.	.	K	.	I	.	.	.	D	.	.	.	.	.	.	.	.	.	.	.	.	.	.	.	.	.	.	.	G	.	.	.
E1620	≤8	AHXF01000060.1	.	.	.	.	.	.	A	.	G	.	.	.	Q	.	.	E	.	.	.	.	.	.	Q	.	.	.	Q	.	.	A	I	.	S	.	.	.	.	.	.	.	.	.	.	.	.	.	.	.	A	.	.	.	.	.	.	.	.	.	.	.	.	.	S	.	.	.	.	.	.	.	.	.	.	.	.	.	.	.	.	.	.	.	K	.	I	.	.	.	D	.	.	.	.	.	.	.	.	.	.	.	.	.	.	.	.	.	.	.	G	.	.	.
HF50104	≤8	AITR01000020.1	.	.	.	.	.	.	A	.	G	.	.	.	Q	.	.	E	.	.	.	.	.	.	Q	.	.	.	Q	.	.	A	I	.	S	.	.	.	.	.	.	.	.	.	.	.	.	.	.	.	A	.	.	.	.	.	.	.	.	.	.	.	.	.	S	.	.	.	.	.	.	.	.	.	.	.	.	.	.	.	.	.	.	.	K	.	I	.	.	.	D	.	.	.	.	.	.	.	.	.	.	.	.	.	.	.	.	.	.	.	G	.	.	.
HF50105	≤8	AITS01000013.1	.	.	.	.	.	.	A	.	G	.	.	.	Q	.	.	E	.	.	.	.	.	.	Q	.	.	.	Q	.	.	A	I	.	S	.	.	.	.	.	.	.	.	.	.	.	.	.	.	.	A	.	.	.	.	.	.	.	.	.	.	.	.	.	S	.	.	.	.	.	.	.	.	.	.	.	.	.	.	.	.	.	.	.	K	.	I	.	.	.	D	.	.	.	.	.	.	.	.	.	.	.	.	.	.	.	.	.	.	.	G	.	.	.
HF50106	≤8	AITT01000026.1	.	.	.	.	.	.	A	.	G	.	.	.	Q	.	.	E	.	.	.	.	.	.	Q	.	.	.	Q	.	.	A	I	.	S	.	.	.	.	.	.	.	.	.	.	.	.	.	.	.	A	.	.	.	.	.	.	.	.	.	.	.	.	.	S	.	.	.	.	.	.	.	.	.	.	.	.	.	.	.	.	.	.	.	K	.	I	.	.	.	D	.	.	.	.	.	.	.	.	.	.	.	.	.	.	.	.	.	.	.	G	.	.	.
HF50203	≤8	AITU01000017.1	.	.	.	.	.	.	A	.	G	.	.	.	Q	.	.	E	.	.	.	.	.	.	Q	.	.	.	Q	.	.	A	I	.	S	.	.	.	.	.	.	.	.	.	.	.	.	.	.	.	A	.	.	.	.	.	.	.	.	.	.	.	.	.	S	.	.	.	.	.	.	.	.	.	.	.	.	.	.	.	.	.	.	.	K	.	I	.	.	.	D	.	.	.	.	.	.	.	.	.	.	.	.	.	.	.	.	.	.	.	G	.	.	.
HF50215	≤8	AITW01000030.1	.	.	.	.	.	.	A	.	G	.	.	.	Q	.	.	E	.	.	.	.	.	.	Q	.	.	.	Q	.	.	A	I	.	S	.	.	.	.	.	.	.	.	.	.	.	.	.	.	.	A	.	.	.	.	.	.	.	.	.	.	.	.	.	S	.	.	.	.	.	.	.	.	.	.	.	.	.	.	.	.	.	.	.	K	.	I	.	.	.	D	.	.	.	.	.	.	.	.	.	.	.	.	.	.	.	.	.	.	.	G	.	.	.
VAN219	≤8	AIUW01000023.1	.	.	.	.	.	.	A	.	G	.	.	.	Q	.	.	E	.	.	.	.	.	.	Q	.	.	.	Q	.	.	A	I	G	S	.	.	.	.	.	.	.	.	.	.	.	.	.	.	.	A	.	.	.	.	.	.	.	.	.	.	.	.	.	.	.	.	.	.	.	.	.	.	.	.	.	.	.	.	.	.	.	.	.	K	.	T	.	.	.	D	.	.	.	.	.	.	.	.	.	.	.	.	.	.	.	.	.	.	.	G	.	.	.
VAN222	≤8	AIUX01000013.1	.	.	.	.	.	.	A	.	G	.	.	.	Q	.	.	E	.	.	.	.	.	.	Q	.	.	.	Q	.	.	A	I	G	S	.	.	.	.	.	.	.	.	.	.	.	.	.	.	.	A	.	.	.	.	.	.	.	.	.	.	.	.	.	.	.	.	.	.	.	.	.	.	.	.	.	.	.	.	.	.	.	.	.	K	.	T	.	.	.	D	.	.	.	.	.	.	.	.	.	.	.	.	.	.	.	.	.	.	.	G	.	.	.
VAN476	≤8	ASEB01000017.1	.	.	.	.	.	.	A	.	G	.	.	.	Q	.	.	E	.	.	.	.	.	.	Q	.	.	.	Q	.	.	A	I	G	S	.	.	.	.	.	.	.	.	.	.	.	.	.	.	.	A	.	.	.	.	.	.	.	.	.	.	.	.	.	.	.	.	.	.	.	.	.	.	.	.	.	.	.	.	.	.	.	.	.	K	.	T	.	.	.	D	.	.	.	.	.	.	.	.	.	.	.	.	.	.	.	.	.	.	.	G	.	.	.
E1258	≤8	AHWT01000005.1	.	.	.	.	.	.	A	.	G	S	.	.	Q	N	.	E	.	.	.	.	.	.	Q	.	.	.	Q	.	.	A	I	.	S	.	.	.	.	.	.	.	.	.	.	.	.	.	.	.	A	.	.	.	.	.	.	.	.	.	.	.	.	.	.	.	.	.	.	.	.	.	E	.	.	.	.	.	.	.	.	.	.	.	K	.	T	.	.	.	D	.	.	.	.	.	.	.	.	.	.	.	.	.	.	.	.	.	.	.	G	.	.	.
E0269	≤8	AHWK01000016.1, AHWK0100001.1	.	.	.	.	S	.	A	.	G	.	.	.	Q	N	.	E	.	.	.	.	.	.	Q	.	.	.	Q	.	.	A	I	.	S	.	.	.	.	.	.	.	.	.	.	.	.	.	.	.	A	.	.	.	.	.	.	.	.	.	.	.	.	.	.	.	.	.	.	.	.	.	.	.	.	.	.	.	.	.	.	.	.	.	K	.	T	.	.	.	D	.	.	.	.	.	.	.	.	.	.	.	.	.	.	.	.	.	.	.	G	.	.	.
E1630	≤8	AHXK01000019.1	.	.	.	.	.	.	A	.	G	.	.	.	Q	N	.	E	.	.	.	.	.	.	Q	.	.	.	Q	.	.	A	I	.	S	.	.	.	.	.	.	.	.	.	.	.	.	I	.	.	A	.	.	.	.	.	.	.	.	.	.	.	.	.	.	.	A	.	.	.	.	.	.	.	.	.	.	.	.	.	.	.	.	.	K	.	T	E	.	.	D	.	.	.	.	.	.	.	.	.	.	.	.	F	.	.	.	.	.	.	G	.	.	.
E1622	≤8	AHXG01000003.1	.	.	.	.	.	.	A	.	G	.	.	.	Q	.	.	E	.	T	.	D	.	.	Q	.	.	.	Q	.	.	A	I	G	S	.	.	.	.	.	.	.	.	.	.	.	.	.	.	.	A	.	.	.	.	.	.	.	.	.	.	.	.	.	.	.	.	.	.	.	.	.	.	.	.	.	.	.	.	.	.	.	.	.	K	.	T	.	.	.	D	.	.	I	.	.	.	.	.	.	.	.	.	.	.	.	.	.	.	.	G	.	S	.
E1071	≤8	ABQI01000087.1	.	.	.	.	.	.	A	T	G	.	.	.	Q	.	.	E	.	T	.	D	.	.	Q	.	.	.	Q	.	.	A	I	G	S	.	.	.	.	.	.	.	.	.	.	.	.	.	.	.	A	.	.	.	.	.	.	.	.	.	.	.	.	.	.	.	.	.	.	.	.	.	.	.	.	.	.	.	.	.	.	.	.	.	K	.	T	.	.	.	D	.	.	.	.	.	.	.	.	.	.	.	.	.	.	.	.	.	.	.	G	.	S	.
E1050	≤8	AHWQ01000005.1	.	.	.	.	.	.	A	.	G	.	.	.	Q	.	.	E	I	T	.	D	.	.	Q	.	.	.	Q	.	.	A	I	G	S	.	.	.	.	.	.	.	.	.	.	.	.	.	.	.	A	.	.	.	.	.	.	.	.	.	.	.	.	.	.	.	.	.	.	.	.	.	.	.	.	.	.	.	.	.	.	.	.	.	K	.	T	.	.	.	D	.	.	.	.	.	.	.	.	.	.	.	.	.	.	.	.	.	.	.	G	.	S	.
E1576	≤8	AHXA01000008.1	.	.	.	.	.	.	A	.	G	.	.	.	Q	.	.	E	.	T	.	D	.	.	Q	.	.	.	Q	.	.	A	I	G	S	.	.	.	.	.	.	.	.	.	.	.	.	.	.	.	A	.	.	.	.	.	.	.	.	.	.	.	.	.	.	.	.	.	.	.	.	.	.	.	.	.	.	.	.	.	.	.	.	.	K	.	T	.	.	.	D	.	.	.	.	.	.	.	.	.	.	.	.	.	.	.	.	.	.	.	G	.	S	.
E1578	≤8	AHXB01000009.1	.	.	.	.	.	.	A	.	G	.	.	.	Q	.	.	E	.	T	.	D	.	.	Q	.	.	.	Q	.	.	A	I	G	S	.	.	.	.	.	.	.	.	.	.	.	.	.	.	.	A	.	.	.	.	.	.	.	.	.	.	.	.	.	.	.	.	.	.	.	.	.	.	.	.	.	.	.	.	.	.	.	.	.	K	.	T	.	.	.	D	.	.	.	.	.	.	.	.	.	.	.	.	.	.	.	.	.	.	.	G	.	S	.
E1623	≤8	AHXH01000005.1	.	.	.	.	.	.	A	.	G	.	.	.	Q	.	.	E	.	T	.	D	.	.	Q	.	.	.	Q	.	.	A	I	G	S	.	.	.	.	.	.	.	.	.	.	.	.	.	.	.	A	.	.	.	.	.	.	.	.	.	.	.	.	.	.	.	.	.	.	.	.	.	.	.	.	.	.	.	.	.	.	.	.	.	K	.	T	.	.	.	D	.	.	.	.	.	.	.	.	.	.	.	.	.	.	.	.	.	.	.	G	.	S	.
VAN342	≤8	AIVA01000020.1	.	.	.	.	.	.	A	.	G	.	.	.	Q	.	.	E	.	T	.	D	.	.	Q	.	.	.	Q	.	.	A	I	G	S	.	.	.	.	.	.	.	.	.	.	.	.	.	.	.	A	.	.	.	.	.	.	.	.	.	.	.	.	.	.	.	.	.	.	.	.	.	.	.	.	.	.	.	.	.	.	.	.	.	K	.	T	.	.	.	D	.	.	.	.	.	.	.	.	.	.	.	.	.	.	.	.	.	.	.	G	.	S	.
VAN332	≤8	AIUY01000015.1	.	.	.	.	.	.	A	.	G	.	.	.	Q	.	.	E	.	T	.	D	.	.	Q	.	.	.	Q	.	.	A	I	G	S	.	.	.	.	.	.	.	.	.	.	.	.	.	.	.	A	.	.	.	.	.	.	.	.	.	.	.	.	.	.	.	.	.	.	.	.	.	.	.	.	.	.	.	.	.	.	.	.	.	K	.	T	.	.	.	D	.	.	.	.	.	.	.	.	.	.	.	.	.	K	.	.	.	.	.	G	.	S	.
VAN345	≤8	AIVB01000020.1	.	.	.	.	.	.	A	.	G	.	.	.	Q	.	.	E	.	T	.	D	.	.	Q	.	.	.	Q	.	.	A	I	G	S	.	.	.	.	.	.	.	.	.	.	.	.	.	.	.	A	.	.	.	.	.	.	.	.	.	.	.	.	.	.	.	.	.	.	.	.	.	.	.	.	.	.	.	.	.	.	.	.	.	K	.	T	.	.	.	D	.	.	.	.	.	.	.	.	.	.	.	.	.	K	.	.	.	.	.	G	.	S	.
E0688	≤8	AHWO01000001.1	.	.	.	.	.	.	A	.	G	.	.	.	Q	.	.	E	.	.	.	.	.	.	Q	.	.	.	Q	.	.	A	I	.	S	.	.	.	.	.	.	.	.	.	.	.	.	.	.	.	A	.	.	.	.	.	.	.	.	.	.	.	.	.	S	.	.	.	.	.	.	.	.	.	.	.	.	.	.	.	.	.	.	.	K	.	I	.	.	.	D	.	.	.	.	.	.	.	.	.	.	.	.	.	.	.	.	.	.	.	G	.	.	.
NRRLB_2354	≤8	CP004063.1	.	.	.	.	.	.	A	.	G	.	.	.	Q	.	.	E	.	T	.	D	.	.	Q	.	.	.	Q	.	.	A	I	G	S	.	.	.	.	.	.	.	S	.	.	.	.	.	.	.	A	.	.	.	.	.	.	.	.	.	.	.	.	.	.	.	.	.	.	.	.	.	.	.	.	.	.	.	.	.	.	.	.	.	K	.	T	.	.	.	D	.	.	.	.	.	.	.	.	.	.	.	.	.	.	.	.	.	.	.	G	.	S	.
E2369	≥16	AHYH01000012.1	.	.	.	.	.	.	A	.	G	.	.	.	Q	.	.	E	.	T	.	D	.	.	Q	.	.	.	Q	.	.	A	I	G	S	.	.	.	.	.	.	.	.	.	.	.	.	.	.	.	A	.	.	.	.	.	.	.	.	.	.	.	.	.	.	.	.	.	H	.	.	.	.	.	.	.	.	.	A	S	.	.	.	A	K	.	T	.	.	.	D	.	.	.	T	.	.	.	.	S	.	.	.	.	.	.	V	Q	L	.	G	.	S	.
Aus0085	≥16	NC_021994.1	.	.	.	.	.	.	A	.	G	.	.	.	Q	.	.	E	.	T	.	D	.	.	Q	.	.	.	Q	.	.	A	I	G	S	.	.	.	.	.	.	.	.	.	.	.	.	.	.	.	A	.	.	.	.	.	.	.	.	.	.	.	.	.	.	.	.	.	H	.	.	.	.	.	.	.	.	.	A	S	.	.	.	A	K	.	T	.	.	.	D	T	.	.	T	.	.	.	.	S	.	.	.	.	.	.	V	Q	L	.	G	.	S	.
E2560	≥16	AHYI01000006.1	.	.	.	.	.	.	A	.	G	.	.	.	Q	.	.	E	.	T	.	D	.	.	Q	.	.	.	Q	.	.	A	I	G	S	.	.	.	.	.	.	.	.	.	.	.	.	.	.	.	A	.	.	.	.	.	.	.	.	.	.	.	.	.	.	.	.	.	H	.	.	.	.	.	.	.	.	.	A	S	.	.	.	A	K	.	T	.	.	.	D	T	.	.	T	.	.	.	.	S	.	.	.	.	.	.	V	Q	L	.	G	.	S	.
U0317	≥16	ABSW01000111.1	.	.	.	.	.	.	A	.	G	.	.	.	Q	.	.	E	.	T	.	D	.	.	Q	.	.	.	Q	.	.	A	I	G	S	.	.	.	.	.	.	.	.	.	.	.	.	.	.	.	A	.	.	.	.	.	.	.	.	.	.	.	.	.	.	.	.	.	H	.	.	.	.	.	.	.	.	.	A	S	.	.	.	A	K	.	T	.	.	.	D	T	.	.	T	.	.	.	.	S	.	.	.	.	.	.	V	Q	L	.	G	.	S	.
D344	≥16	AF362954.1	.	.	.	.	.	.	A	.	G	.	.	.	Q	.	.	E	.	T	.	D	T	.	Q	.	.	.	Q	.	.	A	I	G	S	.	.	.	.	.	.	.	.	.	.	.	.	.	.	.	A	.	.	.	.	.	.	.	.	.	.	.	.	.	.	.	.	.	.	.	.	.	.	.	.	.	.	.	.	.	.	.	.	T	K	.	T	.	.	.	D	.	.	.	.	.	.	.	.	.	.	.	Y	.	.	E	V	.	.	.	G	A	S	.
SN194	≥16	JN208886.1	.	.	.	.	.	.	A	.	G	.	.	.	Q	.	.	E	.	T	.	D	.	.	Q	.	.	.	Q	.	.	A	I	G	S	.	.	.	R	.	.	.	.	.	.	.	.	.	.	.	A	.	.	.	.	.	.	.	.	.	.	.	.	.	.	D	.	.	.	.	.	.	.	.	.	.	.	.	.	S	D	.	S	.	K	L	T	.	.	.	D	.	.	.	.	.	.	.	.	.	.	.	.	.	.	.	V	.	.	.	G	.	S	.
SE97F1	≥16	KC479676.1	.	.	.	.	.	.	A	.	G	.	.	.	Q	.	.	E	.	T	.	D	.	.	Q	.	.	.	Q	.	.	A	I	G	S	.	.	.	.	D	.	.	.	.	.	.	.	.	.	.	A	.	.	.	.	P	.	.	.	.	.	.	.	.	.	D	.	.	.	C	.	.	.	.	.	.	.	K	.	S	.	.	.	A	K	I	T	.	.	.	D	.	.	.	.	.	.	.	.	.	.	.	.	.	.	.	V	.	.	.	G	.	S	.
164306	≥16	JN208882.1	.	.	.	.	.	.	A	.	G	.	.	.	Q	.	.	E	.	.	.	.	.	.	Q	.	.	.	Q	.	.	A	I	.	S	.	.	.	.	.	.	.	.	.	.	.	.	.	.	.	A	.	.	.	.	.	.	R	.	.	.	.	.	.	S	.	.	.	.	.	.	.	.	.	.	.	.	.	.	.	.	.	.	.	K	.	I	.	.	.	D	.	.	.	.	.	.	.	.	.	.	.	.	.	.	.	.	.	H	.	G	.	.	.
H196	≥16	KC479673.1	.	.	.	.	.	.	A	.	G	.	.	.	Q	.	.	E	.	.	.	.	.	.	Q	.	.	.	Q	.	.	A	I	.	S	.	.	.	.	.	.	.	.	.	.	.	.	.	.	.	A	.	.	.	.	.	.	R	.	.	.	.	.	.	S	.	.	.	.	.	.	.	.	.	.	.	.	.	.	.	.	.	.	.	K	.	I	.	.	.	D	.	.	.	.	.	.	.	.	.	.	.	.	.	.	.	.	.	.	.	G	.	.	.
HM1074	≥16	AIUA01000011.1	.	.	.	.	.	.	A	.	G	.	.	.	Q	.	.	E	.	.	.	.	.	.	Q	.	.	.	Q	.	.	A	I	.	S	.	.	.	.	.	.	.	.	.	.	.	.	.	E	.	A	.	.	.	.	.	.	.	.	.	.	.	.	.	S	.	.	.	.	.	.	.	.	.	.	.	.	.	.	.	.	.	.	.	K	.	I	.	.	.	D	.	.	.	.	.	.	.	.	.	.	.	.	.	.	.	.	.	.	.	G	.	.	.
E1636	≥16	ABRY01000062.1	.	.	.	.	.	.	A	.	G	.	.	.	Q	.	.	E	.	.	.	.	.	.	Q	.	.	.	Q	.	.	A	I	.	S	.	.	.	.	.	.	.	.	.	.	.	.	.	.	.	A	.	.	.	.	.	.	.	.	.	.	.	.	.	S	.	.	.	.	.	.	.	.	.	.	.	.	.	.	.	.	.	.	.	K	.	I	.	.	.	D	.	.	.	.	.	.	.	.	.	.	.	.	.	.	.	.	.	.	.	G	.	.	.
HM1072	≥16	AITY01000037.1	.	.	.	.	.	.	A	.	G	.	.	.	Q	.	.	E	.	.	.	.	.	.	Q	.	.	.	Q	.	.	A	I	.	S	.	.	.	.	.	.	.	.	.	.	.	.	.	.	.	A	.	.	.	.	.	.	.	.	.	.	.	.	.	S	.	.	.	.	.	.	.	.	.	.	.	.	.	.	.	.	.	.	.	K	.	I	.	.	.	D	.	.	.	.	.	.	.	.	.	.	.	.	.	.	.	.	.	.	.	G	.	.	.
HM1073	≥16	AITZ01000019.1	.	.	.	.	.	.	A	.	G	.	.	.	Q	.	.	E	.	.	.	.	.	.	Q	.	.	.	Q	.	.	A	I	.	S	.	.	.	.	.	.	.	.	.	.	.	.	.	.	.	A	.	.	.	.	.	.	.	.	.	.	.	.	.	S	.	.	.	.	.	.	.	.	.	.	.	.	.	.	.	.	.	.	.	K	.	I	.	.	.	D	.	.	.	.	.	.	.	.	.	.	.	.	.	.	.	.	.	.	.	G	.	.	.
9439	≥16	X92687.1	.	.	.	.	.	.	A	.	G	.	.	.	Q	.	.	E	.	T	.	D	.	.	Q	.	.	.	Q	.	.	A	I	G	S	.	.	.	.	.	.	.	.	.	.	.	.	.	.	.	A	.	.	.	.	.	P	.	.	.	.	.	.	.	.	.	.	.	.	.	I	.	.	.	.	.	.	.	.	.	.	.	.	.	K	.	T	.	.	.	D	.	.	.	.	.	A	.	.	.	L	.	.	.	.	.	V	.	.	.	G	.	S	.
EFM-1	≥16	X84861.1	-	-	-	-	.	.	A	.	G	.	.	.	Q	.	.	E	.	T	.	D	.	.	Q	.	.	.	Q	.	.	A	I	.	S	.	.	.	.	.	.	.	.	.	.	.	.	.	.	.	A	.	.	.	.	.	.	.	.	.	.	.	.	.	S	.	.	.	.	.	.	.	.	.	.	.	.	.	.	.	.	.	.	T	K	.	I	.	.	.	D	.	.	.	.	.	.	.	.	.	L	.	.	.	.	.	V	.	.	.	G	.	S	.
GEH323_3	≥16	KC479674.1	.	.	.	.	.	.	A	.	G	.	.	.	Q	.	.	E	.	T	.	D	.	.	Q	.	.	.	Q	.	.	A	I	G	S	.	.	.	.	.	.	.	.	.	.	.	.	.	.	.	A	.	.	.	.	.	.	.	.	.	.	.	.	.	.	D	.	.	H	.	.	.	.	.	.	.	.	.	.	.	.	.	.	A	K	I	T	.	.	.	D	.	.	.	.	.	.	.	.	.	L	.	.	.	.	.	V	.	.	.	G	.	S	.
529940	≥16	JN208883.1	.	.	.	.	.	.	A	.	G	.	.	.	Q	.	.	E	.	T	.	D	.	.	Q	.	.	.	Q	.	.	A	I	G	S	.	H	.	R	.	S	.	.	.	.	.	.	.	.	.	A	.	.	.	.	.	.	.	.	.	.	.	.	.	.	.	.	.	.	.	.	.	.	.	.	.	.	.	.	.	.	.	.	A	K	L	T	.	.	.	D	.	.	.	.	.	.	.	.	.	L	.	.	.	.	.	V	.	.	.	G	.	S	.
GEE4_1	≥16	JN208889.1	.	.	.	.	.	.	A	.	G	.	.	.	Q	.	.	E	.	T	.	D	.	.	Q	.	.	.	Q	.	.	A	I	G	S	.	.	.	.	.	S	.	.	.	.	.	.	.	.	.	A	.	.	.	E	.	.	.	.	.	.	.	.	.	.	.	.	.	.	.	.	.	.	.	.	.	.	K	.	.	.	.	.	A	K	.	T	.	.	.	D	.	.	.	.	.	.	.	.	.	L	.	.	.	.	.	V	.	.	.	G	.	S	.
GEE49_1	≥16	JN208884.1	.	.	.	.	.	.	A	.	G	.	.	.	Q	.	.	E	.	T	.	D	.	.	Q	.	.	.	Q	.	.	A	I	G	S	.	.	.	.	.	.	.	.	.	.	.	.	.	.	.	A	.	.	.	.	.	.	.	.	.	.	.	.	.	.	D	.	.	.	.	.	.	.	.	.	.	.	K	.	.	.	.	.	A	K	.	T	.	.	.	D	.	.	.	.	.	.	.	.	.	L	.	.	.	.	.	V	.	.	.	G	.	S	.
SN449	≥16	JN208887.1	.	.	.	.	.	.	A	.	G	.	.	.	Q	.	.	E	.	T	.	D	.	.	Q	.	.	.	Q	.	.	A	I	G	S	.	.	.	.	.	.	.	.	.	.	.	.	.	.	.	A	.	.	.	.	.	.	.	.	.	.	.	.	.	.	D	.	.	.	.	.	.	.	.	.	.	.	K	.	.	.	.	.	A	K	.	T	.	.	.	D	.	.	.	.	.	.	.	.	.	L	.	.	.	.	.	V	.	.	.	G	.	S	.
Aus0004	≥16	NC_017022.1	.	.	.	.	.	.	A	.	G	.	.	.	Q	.	.	E	.	T	.	D	.	.	Q	.	.	.	Q	.	.	A	I	G	S	.	.	.	.	.	.	.	.	.	.	.	.	.	.	.	A	.	.	.	.	.	.	.	.	.	.	.	.	.	.	.	.	.	.	.	.	.	.	.	.	.	.	K	.	.	.	.	.	A	K	.	T	.	.	.	D	.	.	.	.	.	.	.	.	.	L	.	.	.	.	.	V	.	.	.	G	.	S	.
E1731	≥16	AHXO01000011.1	.	.	.	.	.	.	A	.	G	.	.	.	Q	.	.	E	.	T	.	D	.	.	Q	.	.	.	Q	.	.	A	I	G	S	.	.	.	.	.	.	.	.	.	.	.	.	.	.	.	A	.	.	.	.	.	.	.	.	.	.	.	.	.	.	.	.	.	.	.	.	.	.	.	.	.	.	K	.	.	.	.	.	A	K	.	T	.	.	.	D	.	.	.	.	.	.	.	.	.	L	.	.	.	.	.	V	.	.	.	G	.	S	.
E2883	≥16	AHXX01000007.1	.	.	.	.	.	.	A	.	G	.	.	.	Q	.	.	E	.	T	.	D	.	.	Q	.	.	.	Q	.	.	A	I	G	S	.	.	.	.	.	.	.	.	.	.	.	.	.	.	.	A	.	.	.	.	.	.	.	.	.	.	.	.	.	.	.	.	.	.	.	.	.	.	.	.	.	.	K	.	.	.	.	.	A	K	.	T	.	.	.	D	.	.	.	.	.	.	.	.	.	L	.	.	.	.	.	V	.	.	.	G	.	S	.
E4389	≥16	AHYJ01000006.1, AHYJ01000005.1	.	.	.	.	.	.	A	.	G	.	.	.	Q	.	.	E	.	T	.	D	.	.	Q	.	.	.	Q	.	.	A	I	G	S	.	.	.	.	.	.	.	.	.	.	.	.	.	.	.	A	.	.	.	.	.	.	.	.	.	.	.	.	.	.	.	.	.	.	.	.	.	.	.	.	.	.	K	.	.	.	.	.	A	K	.	T	.	.	.	D	.	.	.	.	.	.	.	.	.	L	.	.	.	.	.	V	.	.	.	G	.	S	.
E1293	≥16	AHWU01000013.1	.	.	.	.	.	.	A	.	G	.	.	.	Q	.	.	E	.	T	.	D	.	.	Q	.	.	.	Q	.	.	A	I	G	S	.	.	.	.	.	.	.	.	.	.	.	.	.	.	.	A	.	.	.	.	.	.	.	.	.	.	.	.	.	.	.	.	.	.	.	.	.	.	.	.	.	.	K	.	S	.	.	.	A	K	.	T	.	.	.	D	.	.	.	.	.	.	.	.	.	.	.	.	.	.	.	V	.	.	.	G	.	S	.
E1644	≥16	AHYG01000012.1	.	.	.	.	.	.	A	.	G	.	.	.	Q	.	.	E	.	T	.	D	.	.	Q	.	.	.	Q	.	.	A	I	G	S	.	.	.	.	.	.	.	.	.	.	.	.	.	.	.	A	.	.	.	.	.	.	.	.	.	.	.	.	.	.	.	.	.	.	.	.	.	.	.	.	.	.	K	.	S	.	.	.	A	K	.	T	.	.	.	D	.	.	.	.	.	.	.	.	.	.	.	.	.	.	.	V	.	.	.	G	.	S	.
GESN71_1	≥16	JN208885.1	.	.	.	.	.	.	A	.	G	.	.	.	Q	.	.	E	.	T	.	D	.	.	Q	.	.	.	Q	.	.	A	I	G	S	.	.	.	.	.	.	.	.	.	.	.	.	.	.	.	A	V	.	.	.	.	.	.	.	.	.	.	.	.	.	.	.	.	.	.	.	.	.	.	.	.	.	K	.	S	.	.	.	A	K	.	T	.	.	.	D	.	.	.	.	.	.	.	.	.	.	.	.	.	.	.	V	.	.	.	G	.	S	.
HPH6	≥16	KC479675.1	.	.	.	.	.	.	A	.	G	.	.	.	Q	.	.	E	.	T	.	D	.	.	Q	.	.	.	Q	.	.	A	I	G	S	.	.	.	.	.	.	.	.	.	.	.	.	.	.	.	A	.	.	.	.	.	.	.	.	.	.	.	.	.	.	.	.	.	.	.	.	.	.	.	.	.	.	.	.	.	.	.	.	A	K	L	T	.	.	.	D	.	.	.	.	.	.	.	.	.	L	.	.	.	.	.	V	.	.	.	G	.	S	.
E0333	≥16	AHWL01000001.1	.	.	.	.	.	.	A	.	G	.	.	.	Q	.	.	E	.	T	.	D	.	.	Q	.	.	.	Q	.	.	A	I	G	S	.	.	.	.	.	.	.	.	.	.	.	.	.	.	.	A	.	.	.	.	.	.	.	.	.	.	.	.	.	.	.	.	.	.	.	.	.	.	.	.	.	.	.	.	.	.	.	.	T	K	.	T	.	.	.	D	.	.	.	.	.	.	.	.	.	L	.	.	.	.	.	V	.	.	.	G	.	S	.
E1162	≥16	ABQJ01000017.1	.	.	.	.	.	.	A	.	G	.	.	.	Q	.	.	E	.	T	.	D	.	.	Q	.	.	.	Q	.	.	A	I	G	S	.	.	.	.	.	.	.	.	.	.	.	.	.	.	.	A	.	.	.	.	.	.	.	.	.	.	.	.	.	.	.	.	.	.	.	.	.	.	.	.	.	.	.	.	.	.	.	.	T	K	.	T	.	.	.	D	.	.	.	.	.	.	.	.	.	L	.	.	.	.	.	V	.	.	.	G	.	S	.
GE70411_2	≥16	JN208888.1	.	.	.	.	.	.	A	.	G	.	.	.	Q	.	.	E	.	T	.	D	.	.	Q	.	.	.	Q	.	.	A	I	G	S	.	.	.	.	.	.	.	.	.	.	.	.	.	.	.	A	.	.	.	.	.	.	.	.	.	.	.	.	.	.	.	.	.	.	.	.	.	.	.	.	.	.	.	.	.	.	.	.	T	K	.	T	.	.	.	D	.	.	.	.	.	.	.	.	.	L	.	.	.	.	.	V	.	.	.	G	.	S	.
HM1071	≥16	AITX01000027.1	.	.	.	.	.	.	A	.	G	.	.	.	Q	.	.	E	.	T	.	D	.	.	Q	.	.	.	Q	.	.	A	I	G	S	.	.	.	.	.	.	.	.	.	.	.	.	.	.	.	A	.	.	.	.	.	.	.	.	.	.	.	.	.	.	.	.	.	.	.	.	.	.	.	.	.	.	.	.	.	.	.	.	T	K	.	T	.	.	.	D	.	.	.	.	.	.	.	.	.	L	.	.	.	.	.	V	.	.	.	G	.	S	.
TX0133a01	≥16	AECJ01000122.1	.	.	.	.	.	.	A	.	G	.	.	.	Q	.	.	E	.	T	.	D	.	.	Q	.	.	.	Q	.	.	A	I	G	S	.	.	.	.	.	.	.	.	.	.	.	.	.	.	.	A	.	.	.	.	.	.	.	.	.	.	.	.	.	.	.	.	.	.	.	.	.	.	.	.	.	.	.	.	.	.	.	.	T	K	.	T	.	.	.	D	.	.	.	.	.	.	.	.	.	L	.	.	.	.	.	V	.	.	.	G	.	S	.

TX0133a04	≥16	AEBC01000061.1	
H80721	≥16	X84862.1	
1231410	≥16	ACBA01000014.1	
1231408	≥16	GG688547.1, ACBB01000047.1	
E1133	≥16	AHWR01000016.1	
E4452	≥16	AEOU01000130.1	
E4453	≥16	AEDZ01000364.1	
E1321	≥16	AHYF01000006.1	
E1392	≥16	AHWV01000002.1	
E1679	≥16	ABSC01000284.1	
NEF1	≥16	AXOK01000016.1, AXOK01000017.1	
DO	16	NC_017960.1	
TX0133B	16	AECI01000046.1	
TX0133A	32	AECH01000021.1	
E1904	32	AHXQ01000004.1	
ERV99	>32/R	AMAQ01	
ERV26	>32/R	AMAT01	
ERV28	>32/R	MTRY00000000	
ERV30	>32/R	MTRW00000000	
ERV34	>32/R	MJFW00000000	
ERV35	>32/R	MJFX00000000	
ERV218	64	MJFJ00000000	
ERV223	64	MJFL00000000	
2043	64	Bhavarth	
2036	64	Bhavarth	
2053	64	Bhavarth	
ERV174	64	MJFC00000000	
ERV175	64	MJFD00000000	
ERV177	64	MJFE00000000	
ERV180	64	MJFF00000000	
ERV212	64	MTRX00000000	
ERV214	64	MJFI00000000	
TX0082	64	AEBU01000140.1	
TX0133C	64	AEBG01000194.1	
ERV38	64	AMAS01	
ERV69	64	AMAR01	
505	>64/R	AMBL01000029.1, AMBL01000072.1	
MB871	>64/R	Bhavarth	
UVA5	>64/R	Bhavarth	
MB021	>64/R	Bhavarth	
ERV98	>64/R	MJFY00000000	
C621	>64/R	AMBB01	
ERV102	>64/R	AMAX01	
ERV89	>64/R	MTRT00000000	
MB069	>64/R	Bhavarth	
C11527	>64/R	Bhavarth	
1979	>64/R	Bhavarth	
1987	>64/R	Bhavarth	
2019	>64/R	Bhavarth	
2020	>64/R	Bhavarth	
ERV275	>64/R	MJFS00000000	
ERV280	>64/R	MJFU00000000	
1996	>64/R	Bhavarth	
ERV251	>64/R	MJFP00000000	
UVA8	>64/R	Bhavarth	
UVA7	>64/R	Bhavarth	
S11491	>64/R	Bhavarth	
MB662	>64/R	Bhavarth	
MB613	>64/R	Bhavarth	
MB460	>64/R	Bhavarth	
MB442	>64/R	Bhavarth	
MB278	>64/R	Bhavarth	
MB119	>64/R	Bhavarth	
MB1054	>64/R	Bhavarth	
2045	>64/R	Bhavarth	
2033	>64/R	Bhavarth	
2030	>64/R	Bhavarth	
2027	>64/R	Bhavarth	
2008	>64/R	Bhavarth	
1999	>64/R	Bhavarth	
1994	>64/R	Bhavarth	
1992	>64/R	Bhavarth	
1991	>64/R	Bhavarth	
1982	>64/R	Bhavarth	
1977	>64/R	Bhavarth	
1963	>64/R	Bhavarth	
1958	>64/R	Bhavarth	
1953	>64/R	Bhavarth	
1954	>64/R	Bhavarth	
1955	>64/R	Bhavarth	
1975	>64/R	Bhavarth	
1985	>64/R	Bhavarth	
1988	>64/R	Bhavarth	
1998	>64/R	Bhavarth	
2003	>64/R	Bhavarth	
2007	>64/R	Bhavarth	
2009	>64/R	Bhavarth	
2022	>64/R	Bhavarth	
2040	>64/R	Bhavarth	
2050	>64/R	Bhavarth	
2056	>64/R	Bhavarth	
511	>64/R	AMBH01000038.1	
513	>64/R	AMBG01000061.1	
514	>64/R	AMBF01000038.1	
515	>64/R	AMBE01000026.1	
E422	>64/R	AMAZ01000004.1	
ERV115	>64/R	MTRV00000000	
ERV118	>64/R	MTRU00000000	
ERV133	>64/R	MJEY00000000	
ERV155	>64/R	MJEZ00000000	
ERV157	>64/R	MJFA00000000	
ERV161	>64/R	AMAW01	

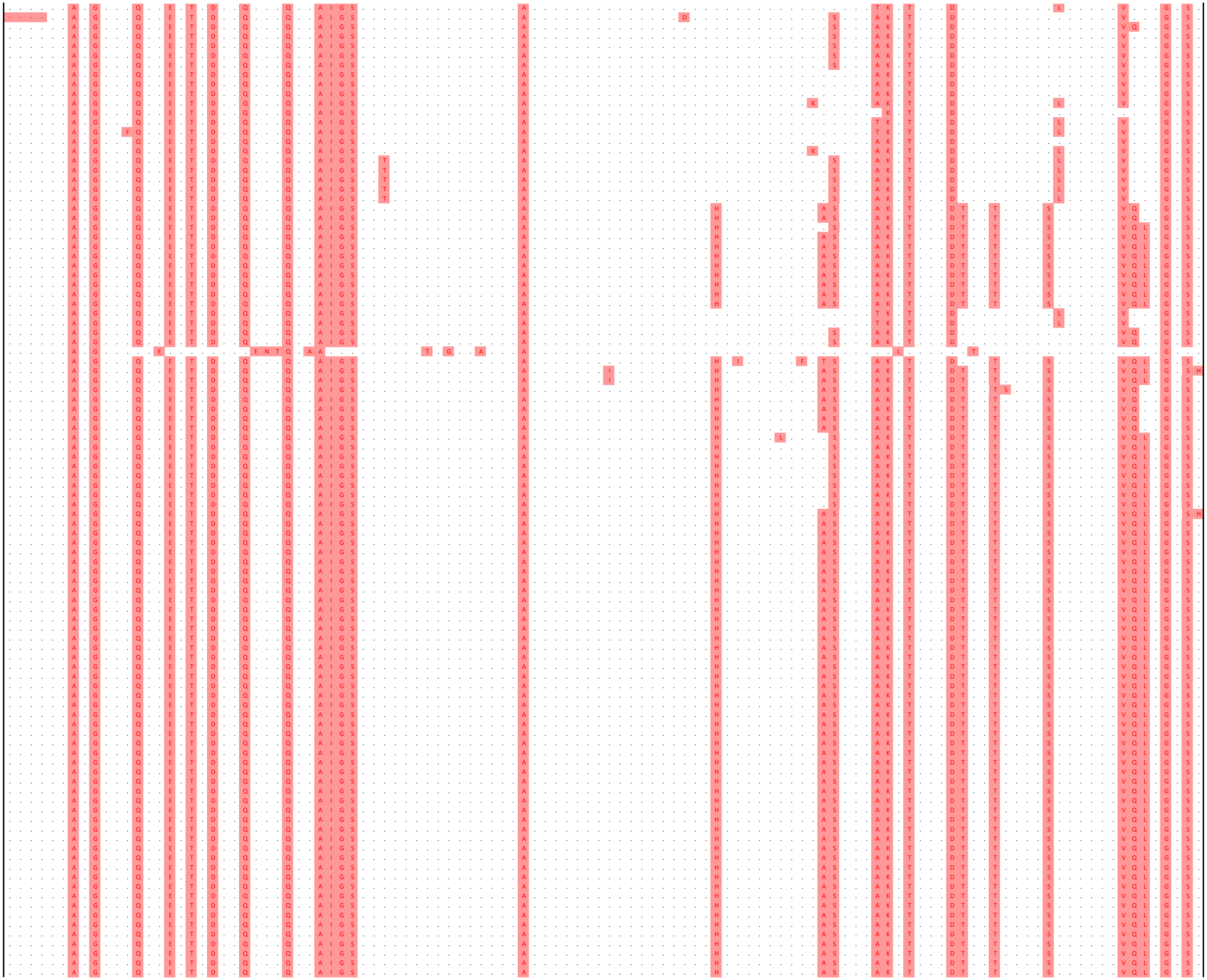

**ERV165**	>64/R	AMAV01	......A.G...Q..E.T.D..Q...Q..AIGS...............A.................H.........AS...AK.T...DT..T....S......VQL.G.S.
**ERV168**	>64/R	AMAU01	......A.G...Q..E.T.D..Q...Q..AIGS...............A.................H.........AS...AK.T...DT..T....S......VQL.G.S.
**ERV172**	>64/R	MJFB00000000	......A.G...Q..E.T.D..Q...Q..AIGS...............A.................H.........AS...AK.T...DT..T....S......VQL.G.S.
**ERV190**	>64/R	MJFG00000000	......A.G...Q..E.T.D..Q...Q..AIGS...............A.................H.........AS...AK.T...DT..T....S......VQL.G.S.
**ERV196**	>64/R	MJFH00000000	......A.G...Q..E.T.D..Q...Q..AIGS...............A.................H.........AS...AK.T...DT..T....S......VQL.G.S.
**ERV220**	>64/R	MJFK00000000	......A.G...Q..E.T.D..Q...Q..AIGS...............A.................H.........AS...AK.T...DT..T....S......VQL.G.S.
**ERV231**	>64/R	MJFM00000000	......A.G...Q..E.T.D..Q...Q..AIGS...............A.................H.........AS...AK.T...DT..T....S......VQL.G.S.
**ERV237**	>64/R	MJFN00000000	......A.G...Q..E.T.D..Q...Q..AIGS...............A.................H.........AS...AK.T...DT..T....S......VQL.G.S.
**ERV242**	>64/R	MJFO00000000	......A.G...Q..E.T.D..Q...Q..AIGS...............A.................H.........AS...AK.T...DT..T....S......VQL.G.S.
**ERV263**	>64/R	MJFQ00000000	......A.G...Q..E.T.D..Q...Q..AIGS...............A.................H.........AS...AK.T...DT..T....S......VQL.G.S.
**ERV270**	>64/R	MJFR00000000	......A.G...Q..E.T.D..Q...Q..AIGS...............A.................H.........AS...AK.T...DT..T....S......VQL.G.S.
**ERV281**	>64/R	MJFV00000000	......A.G...Q..E.T.D..Q...Q..AIGS...............A.................H.........AS...AK.T...DT..T....S......VQL.G.S.
**M11518**	>64/R	Bhavarth	......A.G...Q..E.T.D..Q...Q..AIGS...............A.................H.........AS...AK.T...DT..T....S......VQL.G.S.
**MB020**	>64/R	Bhavarth	......A.G...Q..E.T.D..Q...Q..AIGS...............A.................H.........AS...AK.T...DT..T....S......VQL.G.S.
**MB167**	>64/R	Bhavarth	......A.G...Q..E.T.D..Q...Q..AIGS...............A.................H.........AS...AK.T...DT..T....S......VQL.G.S.
**MB263**	>64/R	Bhavarth	......A.G...Q..E.T.D..Q...Q..AIGS...............A.................H.........AS...AK.T...DT..T....S......VQL.G.S.
**MB271**	>64/R	Bhavarth	......A.G...Q..E.T.D..Q...Q..AIGS...............A.................H.........AS...AK.T...DT..T....S......VQL.G.S.
**MB304**	>64/R	Bhavarth	......A.G...Q..E.T.D..Q...Q..AIGS...............A.................H.........AS...AK.T...DT..T....S......VQL.G.S.
**MB407**	>64/R	Bhavarth	......A.G...Q..E.T.D..Q...Q..AIGS...............A.................H.........AS...AK.T...DT..T....S......VQL.G.S.
**MB443**	>64/R	Bhavarth	......A.G...Q..E.T.D..Q...Q..AIGS...............A.................H.........AS...AK.T...DT..T....S......VQL.G.S.
**MB663**	>64/R	Bhavarth	......A.G...Q..E.T.D..Q...Q..AIGS...............A.................H.........AS...AK.T...DT..T....S......VQL.G.S.
**P1123**	>64/R	JH810542.1, AMAP01000100.1	......A.G...Q..E.T.D..Q...Q..AIGS...............A.................H.........AS...AK.T...DT..T....S......VQL.G.S.
**P1139**	>64/R	AMAN01	......A.G...Q..E.T.D..Q...Q..AIGS...............A.................H.........AS...AK.T...DT..T....S......VQL.G.S.
**P1140**	>64/R	JH808980.1, AMAM01000021.1	......A.G...Q..E.T.D..Q...Q..AIGS...............A.................H.........AS...AK.T...DT..T....S......VQL.G.S.
**P1986**	>64/R	JH808239.1, AMAK01000017.1	......A.G...Q..E.T.D..Q...Q..AIGS...............A.................H.........AS...AK.T...DT..T....S......VQL.G.S.
**R446**	>64/R	JH808025.1, AMAJ01000023.1	......A.G...Q..E.T.D..Q...Q..AIGS...............A.................H.........AS...AK.T...DT..T....S......VQL.G.S.
**R494**	>64/R	AMAI01000028.1	......A.G...Q..E.T.D..Q...Q..AIGS...............A.................H.........AS...AK.T...DT..T....S......VQL.G.S.
**R496**	>64/R	AMAH01000068.1	......A.G...Q..E.T.D..Q...Q..AIGS...............A.................H.........AS...AK.T...DT..T....S......VQL.G.S.
**R497**	>64/R	AMAG01000075.1	......A.G...Q..E.T.D..Q...Q..AIGS...............A.................H.........AS...AK.T...DT..T....S......VQL.G.S.
**R499**	>64/R	AMAF01000040.1	......A.G...Q..E.T.D..Q...Q..AIGS...............A.................H.........AS...AK.T...DT..T....S......VQL.G.S.
**S447**	>64/R	JH806868.1, AMAD01000018.1	......A.G...Q..E.T.D..Q...Q..AIGS...............A.................H.........AS...AK.T...DT..T....S......VQL.G.S.
**T21567**	>64/R	Bhavarth	......A.G...Q..E.T.D..Q...Q..AIGS...............A.................H.........AS...AK.T...DT..T....S......VQL.G.S.
**UVA10**	>64/R	Bhavarth	......A.G...Q..E.T.D..Q...Q..AIGS...............A.................H.........AS...AK.T...DT..T....S......VQL.G.S.
**V689**	>64/R	AMAC01000037.1	......A.G...Q..E.T.D..Q...Q..AIGS...............A.................H.........AS...AK.T...DT..T....S......VQL.G.S.
**504**	>64/R	AMBM01000063.1	......A.G...Q..E......Q...Q..AI.S...............A..K..........S......S............K.I...D...................G...
**506**	>64/R	JH813945.1, AMBK01000250.1	......A.G...Q..E.T.D..Q...Q..AIGS...............A..........................K.....AK.T...D.........L.....V...G.S.
**510**	>64/R	AMBI01000132.1	......A.G...Q..E.T.D..Q...Q..AIGS...............A..........................K.....AK.T...D.........L.....V...G.S.
**C1904**	>64/R	AMBD01	......A.G...Q..E.T.D..Q...Q..AIGS...............A..........................K.....AK.T...D.........L.....V...G.S.
**C497**	>64/R	AMBC01000119.1	......A.G...Q..E.T.D..Q...Q..AIGS...............A..........................K.....AK.T...D.........L.....V...G.S.
**ERV279**	>64/R	MJFT00000000	......A.G...Q..E.T.D..Q...Q..AIGS...............A..........................K.....AK.T...D.........L.....V...G.S.
**MB197**	>64/R	Bhavarth	......A.G...Q..E.T.D..Q...Q..AIGS...............A..........................K.....AK.T...D.........L.....V...G.S.
**P1137**	>64/R	AMAO01000169.1	......A.G...Q..E.T.D..Q...Q..AIGS...............A..........................K.....AK.T...D.........L.....V...G.S.
**P1190**	>64/R	JH808818.1, AMAL01000087.1	......A.G...Q..E.T.D..Q...Q..AIGS...............A..........................K.....AK.T...D.........L.....V...G.S.
**E417**	>64/R	JH813696.1, AMBA01000202.1	......A.G...Q..E.T.D..Q...Q..AIGS...............A............................SD..AK.T...D.........L.....V...G.S.
**ERV127**	>64/R	MJEX00000000	......A.G...Q..E.T.D..Q...Q..AIGS...............A............................SD..AK.T...D.........L.....V...G.S.
**503**	>64/R	JH809465.1, AMBN01000040.1	......A.G...Q..E.T.D..Q...Q..AIGS...............A............................S...AK.T...D.........L.....V...G.S.
**R501**	>64/R	AMAE01000246.1	......A.G...Q..E.T.D..Q...Q..AIGS...............A............................S...AK.T...D...............VQ..G.S.
**509**	>64/R	AMBJ01000257.1	......A.G...Q..E.T.D..Q...Q..AIGS...............A............................S...AK.T...D...............V...G.S.
**ERV113**	>64/R	MJEW00000000	......A.G...Q..E.T.D..Q...Q..AIGS...............A............................S...AK.T...D...............V...G.S.
**1231502**	128	ACAX01	......A.G...Q..E.T.D..Q...Q..AIGS...............A.................H.........AS...AK.T...DT..T....S......VQL.G.S.
**1230933**	128	ACAS01	......A.G...Q..E.T.D..Q...Q..AIGS...............A..........................K.....AK.T...D.........L.....V...G.S.
**E2134**	128	AHXU01	......A.G...Q..E.T.D..Q...Q..AIGS...............A..........................K.S...AK.T...D...............V...G.S.
**E1626**	128	AHXI01	......A.G...Q..E.T.D..Q...Q..AIGS...............A............................SD..AK.T...D.........L.....V...G.S.
**C68**	128	LPUE01	......A.G...Q..E.T.D..Q...Q..AIGS...............A............................S...AKLT...D...............V...G.S.
**ERV1**	256	AMAY01	......A.G...Q..E.T.D..Q...Q..AIGS...............A............................SD..AK.T...D.........L.....V...G.S.

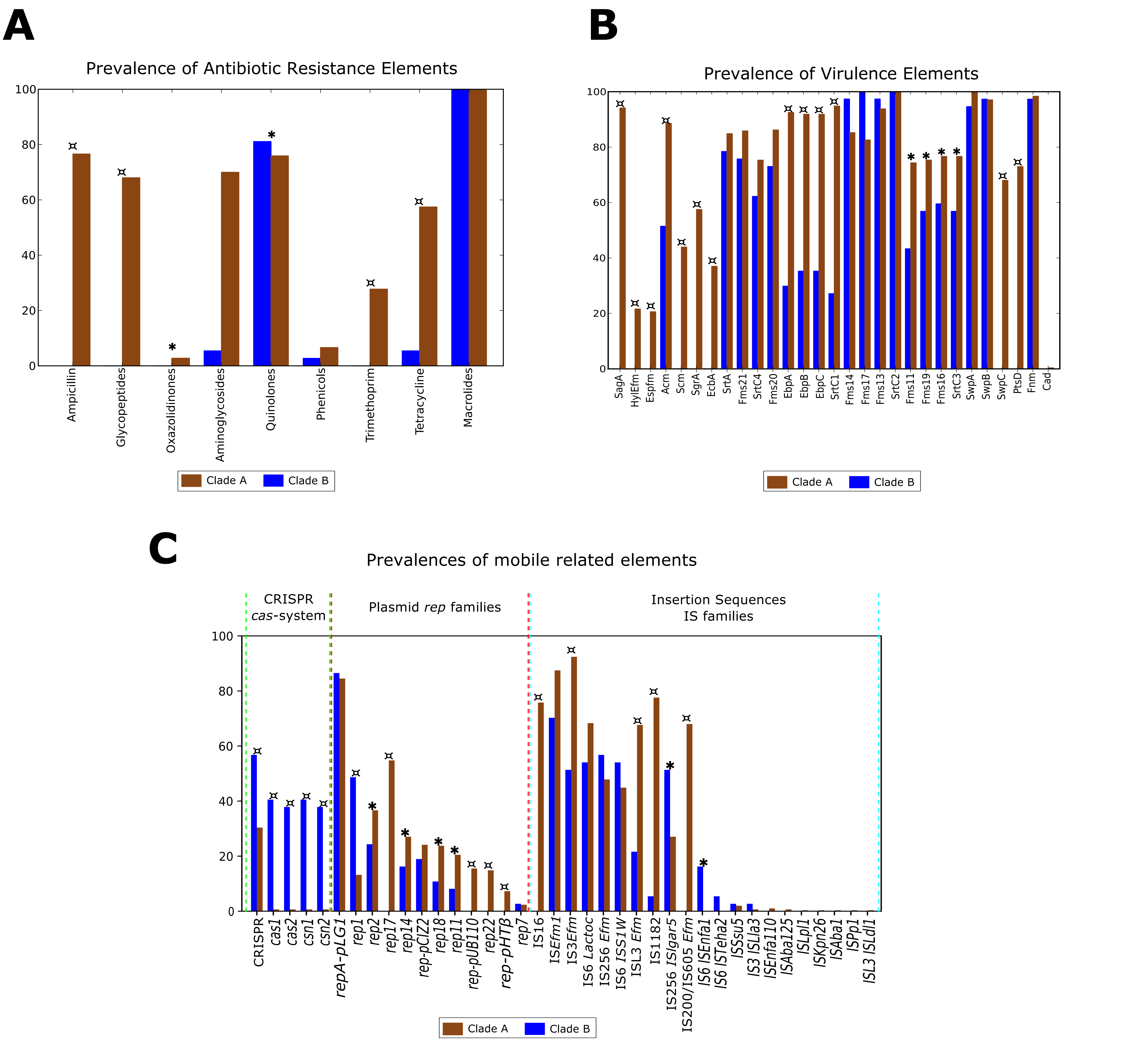

Supplementary Figure 1. (A) Prevalence of genetic elements associated with resistance against different groups of antibiotics in each of the clades. Ampicillin resistance was predicted based on a random forest model using the sequences of the PBP5 protein of susceptible and resistant isolates. (B) Prevalence of putative virulence determinants in each of the clades. (C) Prevalence of mobile genetic elements in each of the clades including isolates harboring the CRISPR cas-system, plasmid rep families and insertion sequences.
✱p-value ≤ 0.05
¤p-value ≤ 0.001

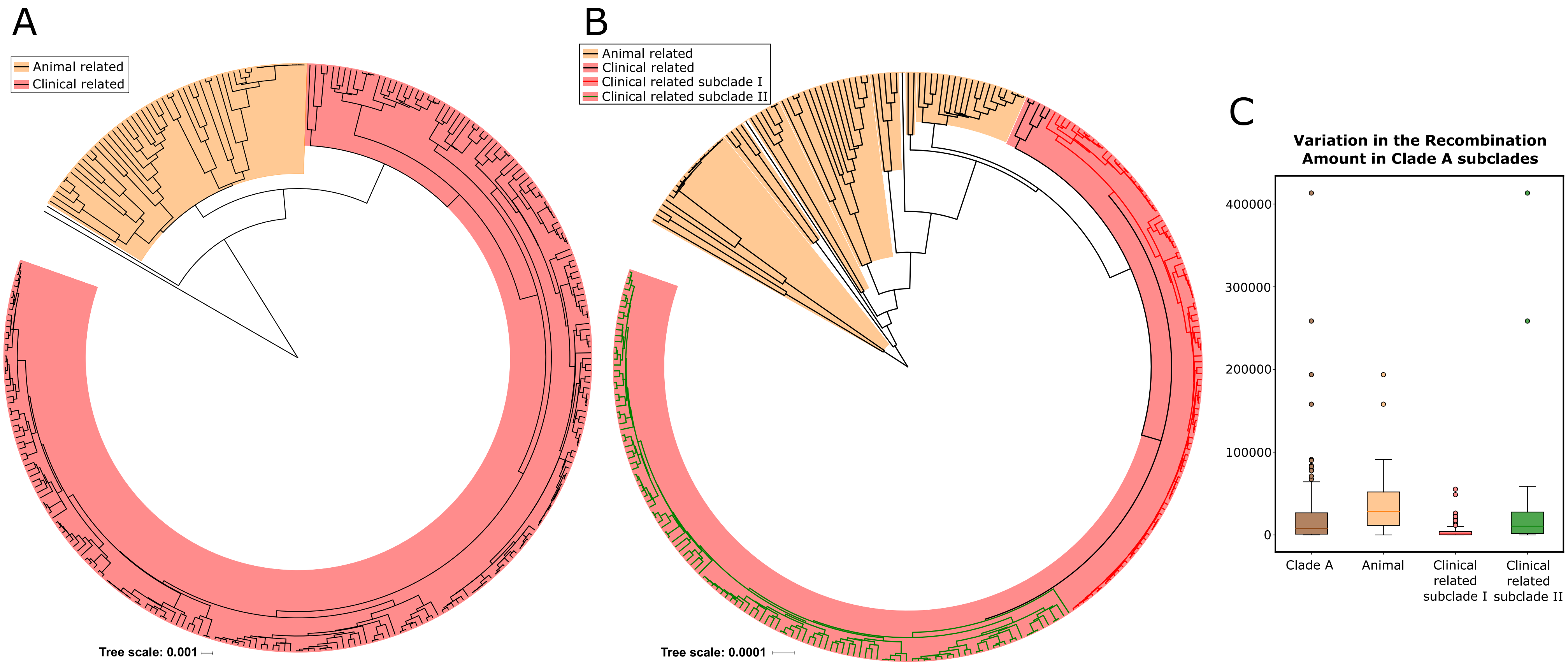

Supplemental Figure 2. Comparison of maximum clade credibility trees including (A) and excluding recombinant regions (B). Both trees show the subclade/branches related to animal (orange background) and clinical sources (red background). (C) Box and whiskers plot of the amount of recombination in the genomes belonging to different subclades/branches in the clade A, based on the recombination regions defined by ClonalFrame. Colored dots represent outliers within each group.

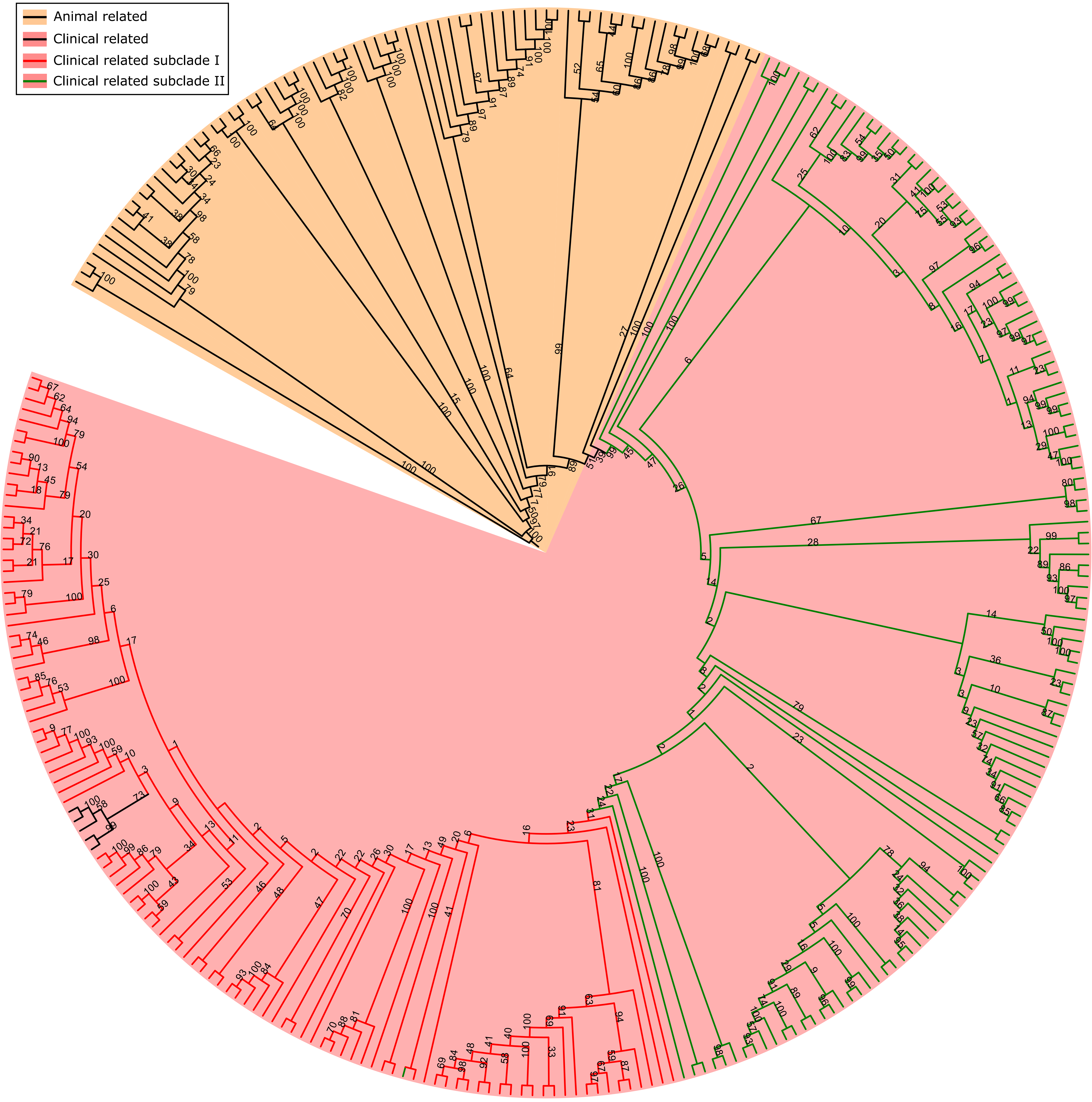

Supplementary Figure 3. Maximum likelihood cladogram of the non recombinant regions of the clade A. Numbers on branches represent the bootstrap support for each branch from 1000 resamplings.

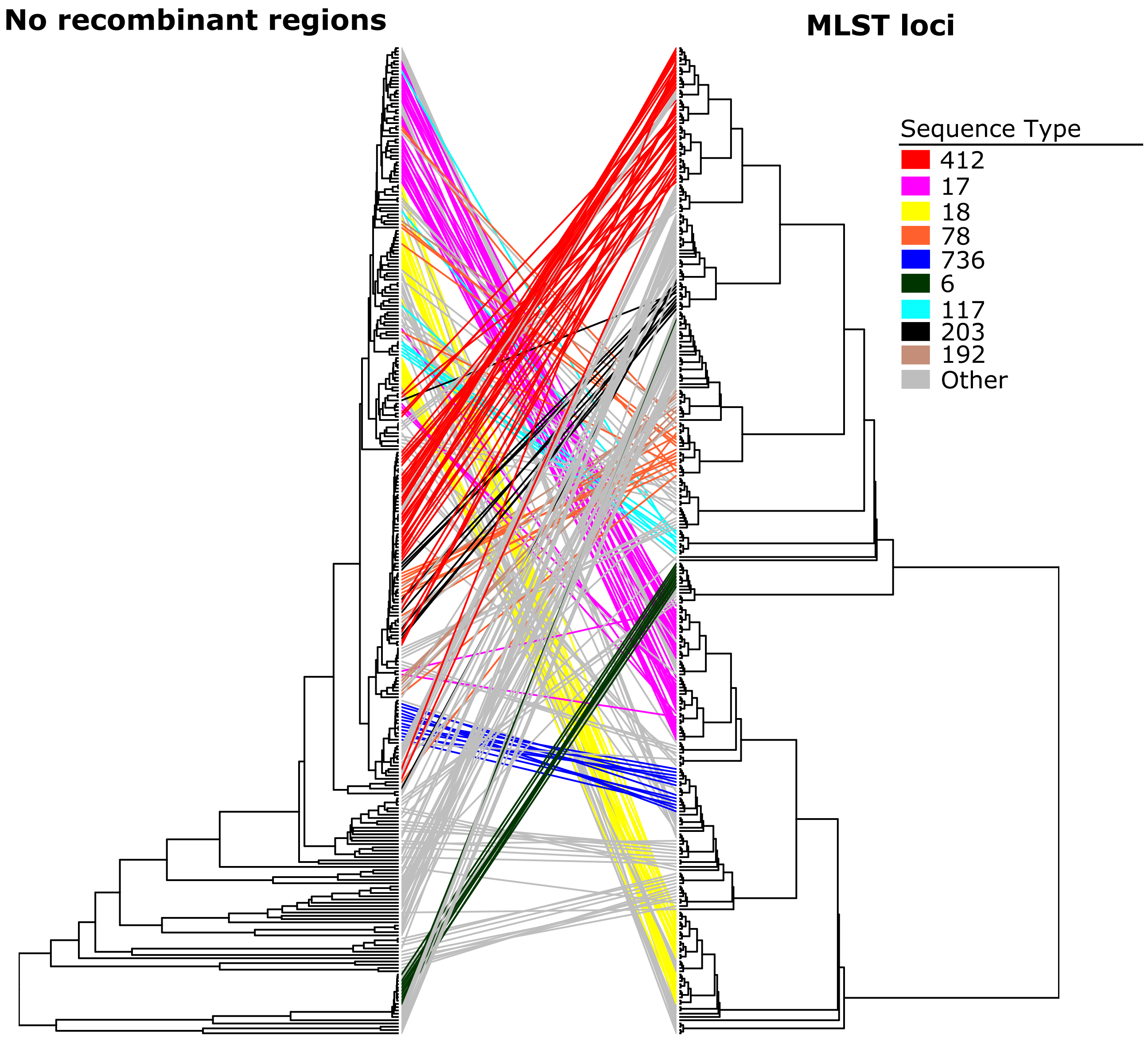

Supplementary figure 4. Tanglegram of phylogenetic reconstructions of Clade A genomes. Tree on the left side is based on non-recombinant regions. Tree on the right side was constructed based on MLST loci. Colored lines in the middle connect the branches from the same genome in the two trees.

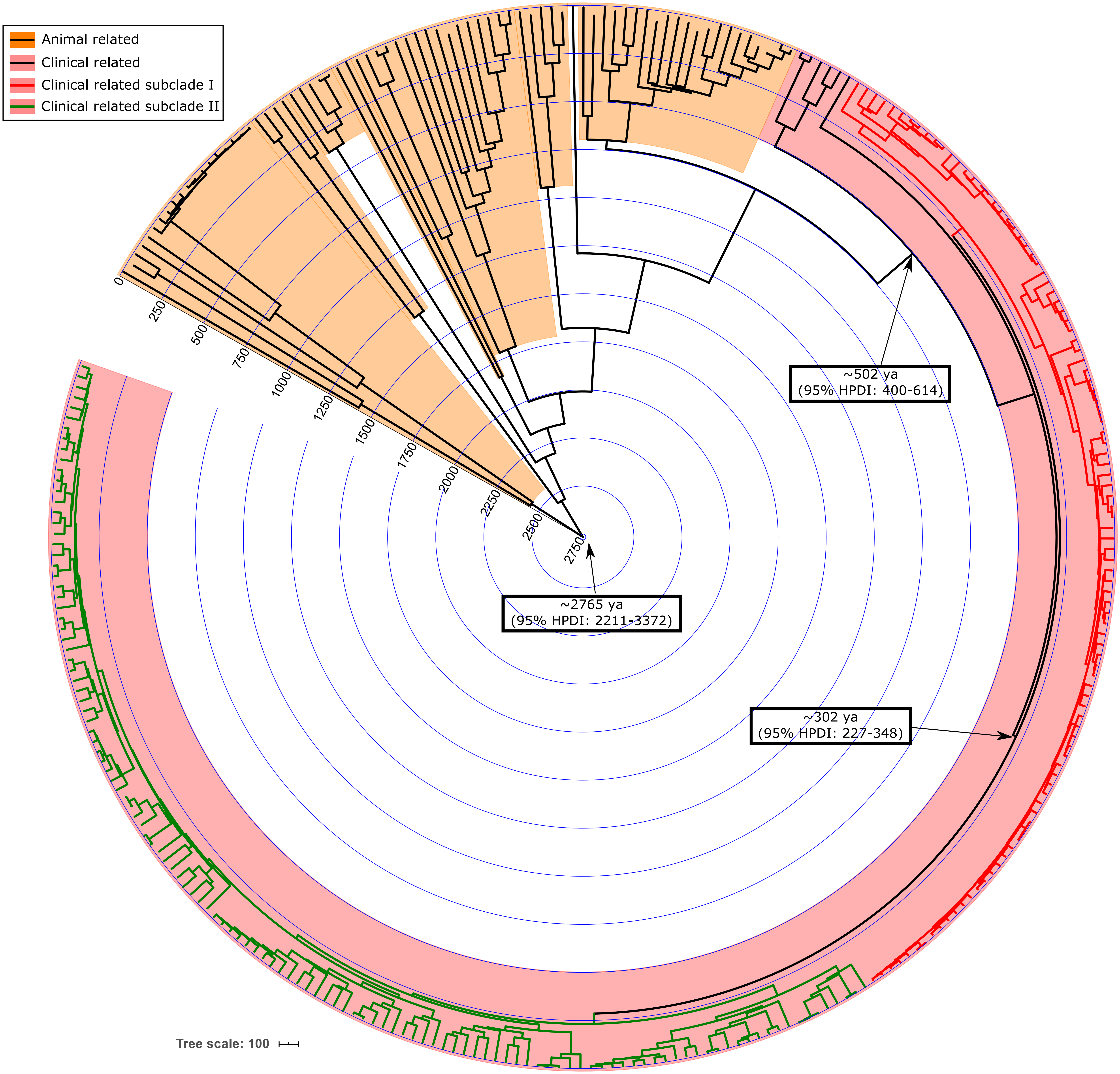

Supplementary Figure 5. Maximum clade credibility tree of the molecular clock analysis of clade A genomes. Each blue ring represents 250 years across branch lengths. Boxes pointing at the splits between major subclades indicate the estimated divergence time between them and their corresponding 95% high probability density intervals (HPDI).

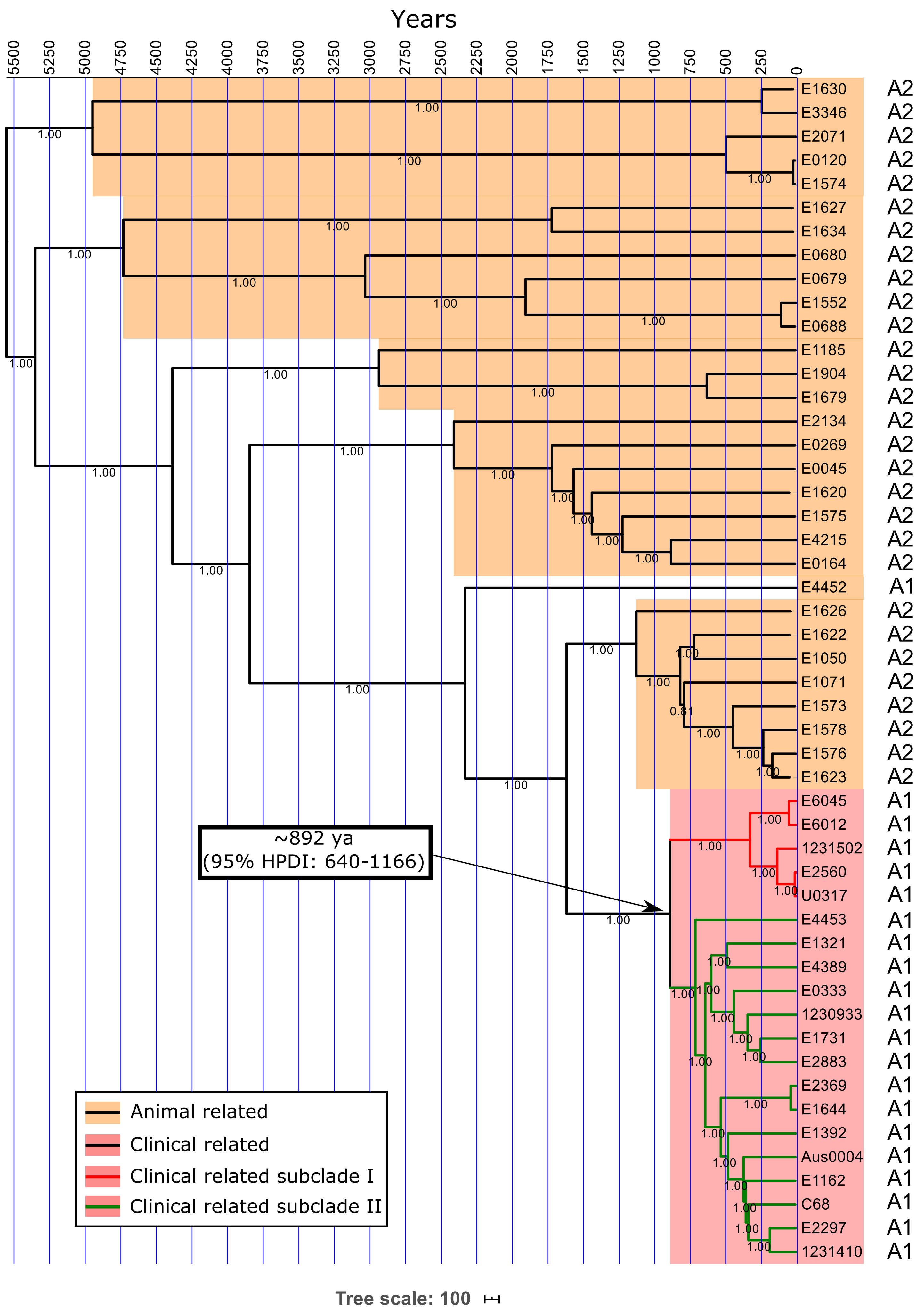

Supplementary Figure 6. Maximum clade credibility tree of the molecular clock of 50 genomes from Clade A (previously described by Lebreton et, al. MBio. 2013). Each blue line represents 250 years across branch lengths. Box pointing the most recent common ancestor for clinical associated isolates and their corresponding 95% high probability density intervals (HPDI). Numbers on the branches shows their posterior probabilities accordingly the Bayesian analysis.
